# Change in physical activity and its association with decline in kidney function: A UK Biobank‐based cohort study

**DOI:** 10.1002/jcsm.13551

**Published:** 2024-08-18

**Authors:** Qiaoling Liu, Carlos Celis‐Morales, Jennifer S. Lees, Naveed Sattar, Frederick K. Ho, Jill P. Pell, Patrick B. Mark, Paul Welsh

**Affiliations:** ^1^ School of Cardiovascular and Metabolic Health University of Glasgow Glasgow UK; ^2^ Human Performance Lab, Education, Physical Activity and Health Research Unit University Católica del Maule Talca Chile; ^3^ School of Health and Wellbeing University of Glasgow Glasgow UK

**Keywords:** Cohort study, Glomerular filtration rate, Kidney function, Physical activity

## Abstract

**Background:**

Previous research on the association between physical activity (PA) and kidney function is inconsistent. The association between muscle mass and serum creatinine (SCr) may have implications for interpreting the effect of PA on estimated glomerular filtration rate (eGFR). Few studies have reported changes in physical activity and changes in kidney function.

**Methods:**

A cohort study was constructed using the UK Biobank. Changes in physical activity were self‐reported as metabolic equivalent task (MET) minutes/week. eGFR was calculated using SCr and cystatin C (CysC). Cox and nonlinear regressions with restricted cubic splines were applied to explore the association between changes in physical activity and rapid decline of kidney function (RDKF, eGFR annual decrease ≥3 mL/min/1.73 m^2^), and the annual change of eGFR. An exploratory analysis of cardiorespiratory fitness as the exposure was conducted.

**Results:**

Among 11 757 participants, the median follow‐up time was 4.4 years. Participants whose PA decreased by 1000 MET minutes/week at the follow‐up assessment had a 2% reduction in risk of developing RDKF_SCr_ (HR = 0.98, 95% CI: 0.96, 1.00). In contrast, a 1000 MET minutes/week increase in PA was associated with a 4% reduction in risk of developing RDKF_CysC_ (HR = 0.96, 95% CI: 0.93, 0.99). A PA increase of 1000 MET minutes/week was associated with eGFR_CysC_ annual increase of 0.04 mL/min/1.73 m^2^ (95% CI: 0.03, 0.06) but no significant changes in eGFR_SCr_.

**Conclusions:**

In this general population study, there are differing associations between changes in PA and changes in kidney function depending on the kidney biomarker used. Increasing PA is modestly associated with improving annual eGFR_CysC_ and reduced risk of RDKF.

## Introduction

While current international physical activity (PA) guidelines recommend engaging in 150 to 300 min of moderate‐intensity physical activity or its equivalent weekly, a significant proportion of adults fail to achieve these recommendations.^1^ The international PA recommendation is backed by substantial evidence that suggests regular physical activity offers multiple health benefits.^2^

Physical activity has a myriad of health benefits and is generally recommend for those with chronic kidney disease (CKD): moderate‐intensity regular exercise recommended for a cumulative duration of at least 150 min/week for patients with high blood pressure (BP) and CKD.^3^ However, these recommendations are mainly based on the cardiovascular benefits of PA, with the impact of exercise on kidney function across a wide range of kidney functions uncertain. Kidney function is currently measured using estimated glomerular filtration rate (eGFR) based on the levels of serum creatinine (SCr), while the use of serum cystatin C (CysC) based eGFR is not widespread in clinical practice. Serum creatinine is a by‐product of muscle metabolism. At normal GFR, muscle mass and function are the most important determinants of eGFR based on serum creatinine (eGFR_SCr_).^4^ Increased physical activity, leading to an increase in muscle mass, could affect kidney function estimates. On the other hand, CysC is freely filtered at the glomerulus and is unaffected by muscle mass, suggesting it may be a more reliable marker of declining kidney function when muscle mass is changeable.^5^, ^6^

Existing research on the association of physical activity with kidney function is inconsistent and often focuses on patients with chronic kidney disease. A meta‐analysis of randomized controlled trials targeting patients with CKD showed that aerobic exercise can reduce serum creatinine levels, improving eGFR_SCr._ ^7^ Patients with CKD may present different associations than the general population. One clinical trial demonstrated that physical activity was inversely associated with the risk of deteriorating kidney function in individuals aged over 65 years, who had an average eGFR_CysC_ of approximately 80 mL/min/1.73 m^2.^ ^8^ However, a similar association was not observed in a younger general population (aged 26–65) with a significantly higher average eGFR_CysC_ of 108 mL/min/1.73 m^2.^ ^9^

While patients with CKD represent a specific subset of the population, the general population consists of a broader demographic. Preventing CKD is critical due to its irreversible nature. Focusing on the broader population, exploring how physical activity affects kidney health in individuals without CKD is vital. This approach could lead to lifestyle guidelines that help maintain kidney function longer. Thus, while managing CKD is crucial, prioritizing research on physical activity's role in kidney health is equally imperative for those not affected by CKD.

Most current cohort studies measure the association between a single baseline value of exposure at entry time and the outcome that occurs by the exit time. A potentially better approach is to use the change in exposure over time. Such results might better elucidate causality.^10^ Here, we studied the impact of changes in physical activity on eGFR; a dynamic approach may better reflect real‐world scenarios.^11^

Furthermore, given that various equations exist for assessing kidney function—each relying on different biomarkers—and that the associations between physical activity and these biomarkers may differ, it is essential to compare outcomes across different eGFR estimating equations. We utilized the extensive data from the UK Biobank to provide simultaneous creatinine and cystatin measurements and its first follow‐up assessment to carry out a cohort study. Our objective is to explore the association between longitudinal changes in physical activity and kidney function in the general population.

## Methods

### Study population

In this research, the study population was drawn from the UK Biobank, a large‐scale population cohort designed to enhance the prevention, diagnosis, and treatment of numerous diseases. Initiated in 2006, UK Biobank recruited around half a million participants, aged 40 to 69 years, from across the UK. A follow‐up assessment was conducted between August 2012 and June 2013, involving approximately 20 000 baseline UK Biobank participants. To exclude participants with possible prevalent CKD, those with baseline eGFR lower than 60 mL/min/1.73 m^2^ were excluded.

### Derivation of the primary exposure

The primary exposure in this study was the change in physical activity among participants between the baseline and the follow‐up assessments. In this study, data on physical activity were collected through a self‐reported questionnaire adapted from the International Physical Activity Questionnaire (IPAQ). Questions like ‘How many minutes did you usually spend doing moderate activities on a typical DAY?’ and ‘In a typical WEEK, on how many days did you walk for at least 10 minutes at a time? (Include walking that you do at work, travelling to and from work, and for sport or leisure)’ were asked. The detailed questionnaire can be assessed online.^12^ The questionnaire categorized physical activity into light, moderate, and vigorous intensity physical activity. Following the IPAQ guideline,^13^ we calculated the metabolic equivalent of task (MET) minutes for each participant in the three intensity categories and summed up the three values to obtain total physical activity expressed as MET minutes/week. Changes in physical activity were estimated by subtracting the total PA at baseline from the total physical activity at follow‐up, 116 and 115 extreme values were replaced by the values at 1st and 99th percentiles respectively.

### Derivation of the secondary exposure

The study's secondary exposure was the change in cardiorespiratory fitness between the baseline and the follow‐up. Cardiorespiratory fitness was assessed in a subset of participants (*n* = 2040) using a 6‐min incremental ramp cycle ergometer test with workload calculated according to age, height, weight, resting heart rate, and sex.^14^ Following validated formulae, cardiorespiratory fitness was presented as maximal oxygen uptake (VO_2_ max, mL/kg/min).^15^

### Study outcomes

The main outcome of this study was the rapid decline of kidney function (RDKF), defined as an annual decrease in eGFR of ≥3/mL/min/1.73 m^2.^ ^16^ The annual change of eGFR was obtained through calculating the differences of eGFRs between the baseline and the follow‐up visit then divided by the follow‐up length in years. We applied three equations published by the CKD Epidemiology Collaboration (CKD‐EPI), including the race‐independent 2021 eGFR_SCr_ equation and eGFR_SCr‐CysC_ equation,^17^ and the 2012 eGFR_CysC_ equation^18^ to calculate eGFR. In the UK Biobank, SCr and CysC were collected and measured by enzymatic analysis on a Beckman Coulter AU5800. The detailed procedure can be found in the published UK Biobank report on laboratory procedures.^19^ The secondary outcome was the average annual change in eGFR, based on two recordings taken at baseline and follow‐up (median 4.41, interquartile interval: 3.68–5.05 years after baseline). For each individual, the change in eGFR between the baseline and the follow‐up assessment was divided by the follow‐up length in years to get the average annual change in eGFR.

### Covariates

Several socio‐demographic, lifestyle and health‐related covariates of this study were selected *a priori* based on published literatures,^8^, ^9^ including sex, race (white, black, South Asian, and other), baseline age (years), Townsend socioeconomic deprivation index, baseline systolic and diastolic blood pressures, baseline body mass index (BMI), smoking status (never/previous/current smoker), self‐reported use of a statin, and self‐reported non‐cancer illness: hypertension, diabetes, chronic heart disease, chronic obstructive pulmonary disease (COPD), stroke, atrial fibrillation, heart failure, and myocardial infarction. More details of how each covariate was assessed can be found in Tables S1–S3.

### Statistical methods

In our study, we first employed Cox regression models to evaluate the impact of changes in self‐reported total physical activity on RDKF, estimating hazard ratios (HRs) and their corresponding 95% confidence intervals (CIs). The entry time was the date of the baseline assessment. The exit time was the date of attending the assessment centre for the first repeat assessment or the date of withdrawal, whichever was earlier. The proportional hazards assumptions were verified using Schoenfeld residuals. To capture potential nonlinear associations, we utilized restricted cubic splines (RCS) with three knots in our Cox regression models. The choice of using only three knots was made as four knots or above lead to overfitting, which could lead to spurious associations and reduce the model's generalizability. The locations of the knots were determined based on Harrell's recommended percentiles. The reference level of RCS models was zero, meaning no exposure changes between the baseline and the follow‐up period. To evaluate the association between changes in physical activity and eGFR, we adopted nonlinear RCS regressions using the same settings as above. As kidney function formulae were different between the sexes, sex‐specific analyses were conducted to explore the possible differences in associations. Since it is possible that a higher baseline PA leads to beneficial renal results (e.g., delay in decline in kidney function) during the follow‐up period, participants were stratified into two groups according to their baseline PA using with a threshold of 1000 MET minutes/week (the upper end of the recommend PA level of current guidelines^20^).

All analyses were adjusted for confounding factors measured at baseline, which included sex, age, Deprivation, systolic and diastolic blood pressure, BMI, smoking status, self‐reported statin use, and self‐reported non‐cancer illnesses (hypertension, diabetes, chronic heart disease, COPD, stroke, atrial fibrillation, heart failure, and myocardial infarction). In the Cox‐regression analyses, age and BMI were incorporated as time‐varying covariates.^21^ All statistical analyses were performed using STATA MP 17.0 (Texas, USA).

## Results

**Figure 1:**
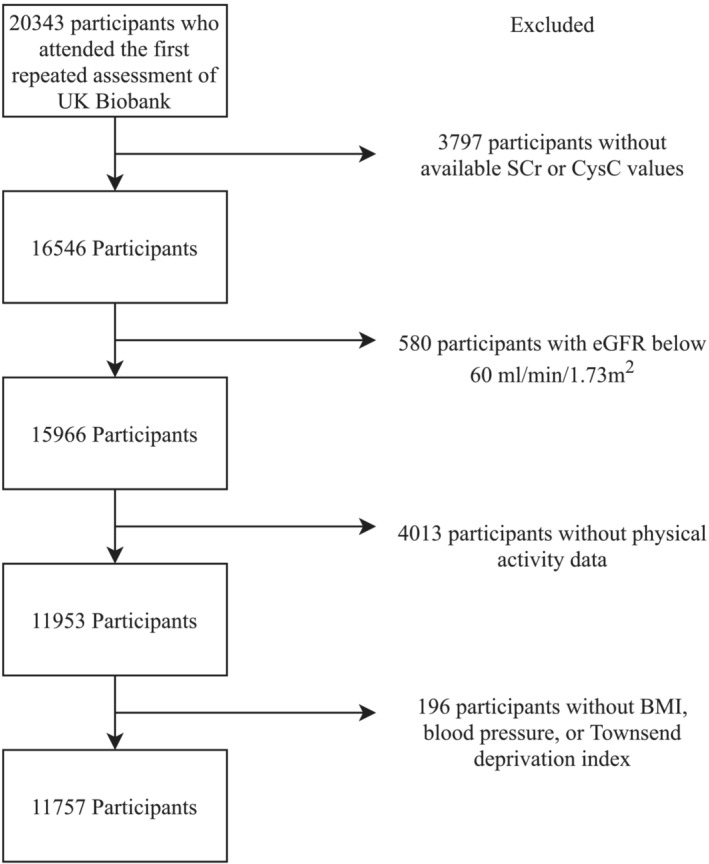
Study flowchart.

A total of 20 343 individuals participated in the first repeated assessment, of whom 16 546 had baseline SCr and CysC data available. After retaining those with eGFR results greater than 60 mL/min/1.73 m^2^, 15 966 individuals remained. After excluding those lacking data on physical activity and covariates, 11 757 individuals were finally included in the study. Among them, 2040 participants also had cardiorespiratory fitness data (Figure 1).

In the 11 757 participants, the median follow‐up time was 4.4 years (interquartile range, [IQR] 1.25 years). At follow‐up, 77.6% (*n* = 9118) had a decline in eGFR_SCr_, and 71.3% (*n* = 8378) had a decline in eGFR_CysC_. The mean (standard deviation) eGFR_SCr_ and eGFR_CysC_ were 95.4 (10.9) and 91.3 (13.2) mL/min/1.73 m^2,^ respectively, at baseline; and fell to 90.6 (11.9) and 87.7 (14.9) mL/min/1.73 m^2^ at the follow‐up assessment. There were more males (52.16% vs. 47.46%) and lower C‐reactive protein (1.03 vs. 1.24 mg/L) among participants with baseline PA ≥ 1000 MET minutes/week versus <1000 MET minutes/week, but few other substantial differences were found comparing these groups (Table S4).

At follow‐up, roughly half of the study population (49.2%, 5789 participants) had decreased their total PA, with a median change of −1095 MET minutes/week (IQR: 2004 MET minutes/week). A total of 5736 participants (48.8%) had increased their total PA over follow‐up, with a median change of 1065 MET minutes/week (IQR: 1823 MET minutes/week). The remaining 232 individuals reported no change in their total PA levels. At the follow‐up visit, more participants had developed co‐morbidities or were using cholesterol lowering medication than at baseline. The median (IQR) annual change of eGFR_SCr_ and eGFR_CysC_ was −0.92 (1.90) and −0.88 (1.75) mL/min/1.73 m^2^, respectively (Table S5).

More individuals were identified as having RDKF at follow‐up using eGFR_SCr_ than eGFR_CysC_ (1731 vs. 1285 participants). Participants with RDKF_SCr_ were less likely to be male (50.2% vs. 57.9%), have hypertension (52.9% vs. 58.8%), use a statin (14.2% vs. 19.3%), and smaller median of C‐reactive protein (1.14 mg/L vs. 1.26 mg/L) compared those with RDKF based on eGFR_CysC_ (Table 1).

**Table 1:** Baseline characteristics of the study population, stratified by the status of RDKF at the end of follow‐up period

	All	RDKF by eGFR_SCr_		RDKF by eGFR_CysC_	
		Yes	No	Yes	No
Sample size, *n* (%)^a^	11 757 (100)	1731 (14.72)	10 026 (85.28)	1285 (10.93)	10 472 (89.07)
Age, years	57.25 (7.35)	57.26 (7.34)	57.25 (7.35)	58.79 (6.93)	57.06 (7.38)
Male, *n* (%)	5978 (50.85)	869 (50.20)	5109 (50.96)	744 (57.90)	5234 (49.98)
Ethnicity, *n* (%)					
White	11 530 (98.07)	1701 (98.27)	9829 (98.04)	1259 (97.98)	10 271 (98.08)
Black	47 (0.40)	6 (0.35)	41 (0.41)	3 (0.23)	44 (0.42)
South Asian	61 (0.52)	6 (0.35)	55 (0.55)	9 (0.70)	52 (0.50)
Others	119 (1.01)	18 (1.04)	101 (1.01)	14 (1.09)	105 (1.00)
Body mass index, kg/m^2^	26.46 (4.13)	26.74 (4.18)	26.41 (4.12)	27.13 (4.37)	26.38 (4.09)
Smoking, *n* (%)					
Never	7030 (59.79)	1037 (59.91)	5993 (59.77)	735 (57.20)	6295 (60.11)
Previous	4026 (34.24)	582 (33.62)	3444 (34.35)	460 (35.80)	3566 (34.05)
Current	701 (5.96)	112 (6.47)	589 (5.87)	90 (7.00)	611 (5.83)
Townsend deprivation index, median (IQR)	−2.76 (3.13)	−2.83 (3.18)	−2.76 (3.12)	−2.69 (3.12)	−2.77 (3.12)
Systolic blood pressure, mmHg	137.72 (18.15)	139.60 (18.94)	137.40 (17.99)	141.43 (18.85)	137.26 (18.01)
Diastolic blood pressure, mmHg	81.52 (9.79)	82.33 (10.07)	81.38 (9.74)	82.28 (10.12)	81.43 (9.75)
Serum creatinine, mg/dL	0.81 (0.15)	0.81 (0.13)	0.81 (0.15)	0.83 (0.15)	0.81 (0.15)
Serum cystatin C, mg/L	0.87 (0.11)	0.89 (0.11)	0.87 (0.11)	0.88 (0.09)	0.87 (0.11)
C‐reactive protein, mg/L, median (IQR)	1.08 (1.64)	1.14 (1.74)	1.08 (1.60)	1.26 (1.72)	1.06 (1.61)
Estimated glomerular filtration rate, mean (SD)					
eGFR_SCr_, mL/min/1.73 m^2^	95.44 (10.90)	96.50 (8.91)	95.25 (11.19)	93.67 (10.95)	95.65 (10.87)
eGFR_CysC_, mL/min/1.73 m^2^	91.28 (13.24)	89.65 (13.16)	91.56 (13.23)	90.50 (11.14)	91.37 (13.47)
Co‐morbidities, *n* (%)					
Atrial fibrillation	79 (0.67)	17 (0.98)	62 (0.62)	18 (1.40)	61 (0.58)
Chronic obstructive pulmonary disease	99 (0.84)	20 (1.16)	79 (0.79)	13 (1.01)	86 (0.82)
Coronary heart disease	365 (3.10)	69 (3.99)	296 (2.95)	66 (5.14)	299 (2.86)
Diabetes	405 (3.44)	79 (4.56)	326 (3.25)	81 (6.30)	324 (3.09)
Heart failure	6 (0.05)	2 (0.12)	4 (0.04)	0 (0.00)	6 (0.06)
Hypertension	5867 (49.90)	916 (52.92)	4951 (49.38)	756 (58.83)	5111 (48.81)
Myocardial infarction	199 (1.69)	36 (2.08)	163 (1.63)	31 (2.41)	168 (1.60)
Stroke	156 (1.33)	21 (1.21)	135 (1.35)	10 (0.78)	146 (1.39)
Use of statin, *n* (%)	1632 (13.88)	246 (14.21)	1386 (13.82)	248 (19.30)	1384 (13.22)
Total MET minutes/week of physical activity, median (IQR)	1896 (2723)	1848 (2602)	1904 (2755)	1893 (3048)	1898 (2688)
VO_2_max, mL/kg/min, median (IQR)^b^	38.19 (73.87)	37.40 (76.18)	38.59 (73.30)	37.32 (63.06)	38.62 (76.11)

% is the column percentage unless otherwise specified.

CysC, cystatin C; eGFR, estimated glomerular filtration rate; IQR, interquartile range; MET, metabolic equivalent task; RDKF, rapid decline of kidney function; SCr, serum creatinine; SD, standard deviation.

^a^ Row percentage is presented.

^b^ Sample size = 2040 participants.

### Association between changes in total physical activity and rapid decline of kidney function incidence

**Figure 2:**
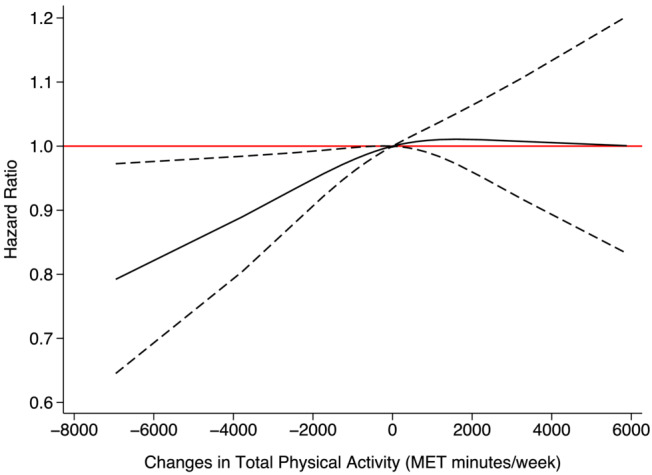
Association between changes in physical activity and RDKF incidence using eGFR_SCr_. The solid black line represents the regression line. Dashed lines on either side of the solid black line show the 95% confidence interval (CI). The red line is for easy reference, and a 95% CI below or above the line is regarded as a meaningful association. Adjusted for sex, race, baseline age, smoking, body mass index, Townsend deprivation index, baseline systolic blood pressure, baseline diastolic pressure, use of statin, hypertension, diabetes, coronary heart disease, chronic obstructive pulmonary disease, stroke, atrial fibrillation, heart failure, and myocardial infarction.

The association between changes in total physical activity and RDKF was observed to yield almost opposite results depending on the kidney function biomarkers used. Using eGFR_SCr_ to ascertain RDKF, the risk decreased as physical activity levels declined. Compared with individuals with consistent physical activity levels from baseline to follow‐up, those who reduced their weekly physical activity by 1000 MET‐min/week over follow‐up had a 2% lower risk of developing RDKF (HR = 0.98, 95% CI: 0.96, 1.00). A reduction of 2000 MET‐min/week decreased the risk further by 5% (HR = 0.95, 95% CI: 0.91, 0.99) (Figure 2). There was no interaction by sex (*P*‐value = 0.75) (Figure S1a,b).

**Figure 3:**
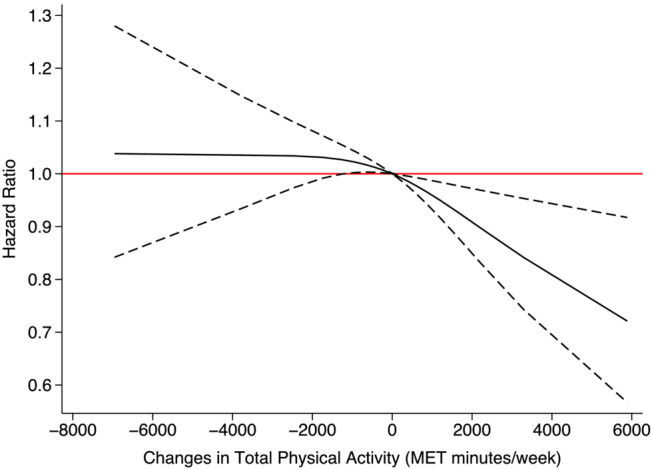
Association between changes in physical activity and RDKF incidence using eGFR_CysC_. The solid black line represents the regression line. Dashed lines on either side of the solid black line show the 95% confidence interval (CI). The red line is for easy reference, and a 95% CI below or above the line is regarded as a meaningful association. Adjusted for sex, race, baseline age, smoking, body mass index, Townsend deprivation index, baseline systolic blood pressure, baseline diastolic pressure, use of statin, hypertension, diabetes, coronary heart disease, chronic obstructive pulmonary disease, stroke, atrial fibrillation, heart failure, and myocardial infarction.

Using eGFR_CysC_, there was a decrease in the incidence of RDKF as physical activity levels rose. Specifically, individuals who increased their weekly physical activity by 1000 MET minutes/week (equivalent to walking 264 min/week) at the follow‐up, compared with those with stable activity levels from baseline, experienced a 4% reduction in the risk of developing RDKF (HR = 0.96, 95% CI: 0.93, 0.99). An increase of 2000 MET minutes/week (equivalent to walking 528 min/week) was associated with to a 9% reduction in risk (HR = 0.91, 95% CI: 0.85, 0.97) (Figure 3). There was no evidence of a sex‐interaction (*P*‐value = 0.17) (Figures S2a,b). There were no associations of changes in total physical activity with RDKF incidence measured by eGFR_SCr‐CysC_ as the outcome (Figure S3a–c).

A total of 3285 participants (27.94%) had baseline PA below the 1000 MET minutes/week threshold. The association of changes in total physical activity with RDKF ascertained by eGFR_SCr_, eGFR_CysC_, and eGFR_SCr‐CysC_ was explored in both strata (above and below 1000 MET minutes/week). No interactions by baseline PA categories for RDKF ascertained by eGFR_SCr_ (*P*‐value = 0.63) and eGFR_SCr‐CysC_ (*P*‐value = 0.37), and a marginal interaction was observed when RDKF ascertained by eGFR_CysC_ (*P*‐value = 0.06) (Figures S1c,d, S2c,d, S3d,e).

### Association between changes in total physical activity and annual change in estimated glomerular filtration rate

**Figure 4:**
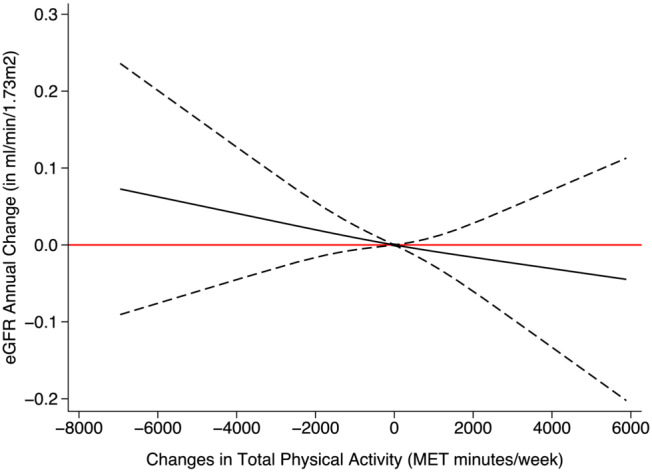
Association between changes in physical activity and the annual change of eGFR_SCr_. The solid black line represents the regression line. Dashed lines on either side of the solid black line show the 95% confidence interval (CI). The red line is for easy reference, and a 95% CI below or above the line is regarded as a meaningful association. Adjusted for sex, race, baseline age, smoking, body mass index, Townsend deprivation index, baseline systolic blood pressure, baseline diastolic pressure, use of statin, hypertension, diabetes, coronary heart disease, chronic obstructive pulmonary disease, stroke, atrial fibrillation, heart failure, and myocardial infarction.

**Figure 5:**
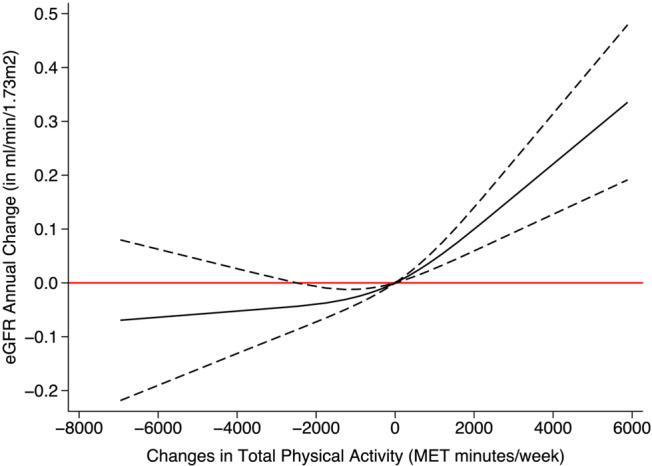
Association between changes in physical activity and the annual change of eGFR_CysC_. The solid black line represents the regression line. Dashed lines on either side of the solid black line show the 95% confidence interval (CI). The red line is for easy reference, and a 95% CI below or above the line is regarded as a meaningful association. Adjusted for sex, race, baseline age, smoking, body mass index, Townsend deprivation index, baseline systolic blood pressure, baseline diastolic pressure, use of statin, hypertension, diabetes, coronary heart disease, chronic obstructive pulmonary disease, stroke, atrial fibrillation, heart failure, and myocardial infarction.

No association was observed between the change in physical activity and the annual change of eGFR_SCr_ (Figure 4, Figure S4a,b). However, there was a highly consistent association with the annual change of eGFR_CysC_ (Figure 5, Figure S5a,b).

In both the overall and sex‐specific analyses, individuals who exhibited an increase in total PA, compared with those who maintained consistent levels from baseline to follow‐up, had a small but statistically significant increase in eGFR_CysC_. Within the general population, individuals whose PA rose by 1000 MET‐min/week from baseline had an eGFR_CysC_ increase of 0.04 mL/min/1.73 m^2^ (coefficient = 0.04, 95% CI: 0.03, 0.06). After additional adjustment for baseline and change in body fat and C‐reactive protein, the association was slightly attenuated (coefficient = 0.03, 95% CI: 0.01, 0.05). For those with a total PA increment of 2000 MET‐min/week, the increase was 0.10 mL/min/1.73 m^2^ (coefficient = 0.10, 95% CI: 0.06, 0.14).

Test for interaction showed significant sex difference (*P*‐value = 0.02). Notably, among men, a decrease in total physical activity levels from baseline was correlated with a negative annual change in eGFR_CysC_. Specifically, men who reduced their physical activity by 1000 MET minutes/week had an annual eGFR_CysC_ decline of 0.04 mL/min/1.73 m^2^ (coefficient = −0.04, 95% CI: −0.06, −0.02). For those with a decrease of 2000 MET minutes/week, the annual change in eGFR_CysC_ was lowered by 0.07 mL/min/1.73 m^2^ (coefficient = −0.07, 95% CI: −0.12, −0.03). Associations between changes in total physical activity and eGFR_SCr‐CysC_ were also identified, with strong similarity to the regression results using eGFR_CysC_ (Figure S6a–c). In participants with baseline PA ≥ 1000 vs. <1000 MET minutes/week, no significant interaction effect was found (Figures S4c,d, S5c,d, and S6d,e).

### Association between changes in cardiorespiratory fitness and markers of kidney function

There was no association between change in fitness levels measured by VO_2_max and the onset of RDKF, irrespective of the kidney function biomarkers considered (Figures S7a–c and S8a–c). While there was no significant link between fitness and the annual change rate of eGFR_SCr_, a modest decrease in the annual change of eGFR_CysC_ was noted among men when their fitness levels decreased from baseline.

In men, for a decrease of 20 mL/kg/min in fitness, the annual eGFR_CysC_ showed an average decline of 0.02 (coefficient = −0.02, 95% CI: −0.04, −0.004). When the fitness reduction reached 30 mL/kg/min, the yearly eGFR_CysC_ decreased by an average of 0.04 (coefficient = −0.04, 95% CI: −0.06, −0.01) (Figures S9a–c and S10a–c).

## Discussion

The study findings suggest that changes in physical activity are associated with changes in kidney function. Notably, the research results differed depending on whether SCr or CysC was used to calculate kidney function. Using SCr to assess kidney function, a decrease in physical activity correlated with lower RDKF incidence, and there were no overall associations of change in PA with change in eGFR_SCr_. In contrast, when using CysC to measure kidney function, increased physical activity was associated with lower RDKF incidence and increase in PA was associated with modest increases in eGFR_CysC_ (with a sex‐interaction). There was no evidence that baseline PA had any effect on the association between changes in PA and the kidneys.

A study of 199 421 Taiwanese individuals with normal kidney function reported that a higher level of habitual physical activity was linked to a lesser decline in eGFR_SCr_ levels and a reduced risk of developing CKD.^22^ Our analyses add to this showing that changes in physical activity may be associated with changes in kidney function, although change in eGFR_CysC_ may be a more optimal way to measure eGFR. Therefore, if these associations are causal, there may be an opportunity for interventions that improve kidney health.

Physical activity, especially leisure‐time physical activity (LTPA), has been examined for its dose–response relationship with various health conditions. A meta‐analysis of LTPA highlighted a negative nonlinear association with incident metabolic syndrome (MetS), whereby 150 min of moderate PA per week (10 MET hours/week) confers a 10% decrease in the risk of MetS, a 20% decrease with doubled PA per week (20 MET hours/week), and a 53% decrease with 70 MET hours/week, compared with inactive participants.^23^ Because MetS and related metabolic perturbances such as type 2 diabetes are established risk factors for CKD, this dose–response association indicates that higher levels of physical activity might be mechanistically related to kidney function.^24^ One study using 7988 Korean participants also revealed that high physical activity can alleviate the risk for chronic kidney disease regardless of sedentary time.^25^

The inverse association between PA and kidney biomarkers may also be explained by the effect of PA on lowering blood pressure. One meta‐analysis has revealed a linear 6% reduction in the risk of hypertension for each 600 MET minutes/week increase in LTPA.^26^ Blood pressure lowering is known to be associated with improved eGFR and reduced development of end‐stage renal disease.^27^

The underlying mechanisms by which physical activity could potentially benefit the kidney may also lie in the effect of PA on reducing adiposity, thus easing inflammation, decreasing oxidative stress, and improving endothelial function. These physiological processes are influenced by adipocytokines, which are known to contribute to obesity‐related complications, including those impacting kidney health.^28^

Several studies have investigated eGFR_SCr_ and eGFR_CysC_ within identical populations, consistently finding that eGFR_SCr_ is less efficient than eGFRC_ysC_ in evaluating health outcomes.^5^, ^29^ Our findings align with this observation. Moreover, when comparing eGFR_SCr_ with directly measured GFR (via Tc99m DTPA plasma clearance), indications suggest that eGFR_SCr_ might bear an intrinsic bias related to muscle mass.^4^ Creatinine's primary internal origin stems from muscle metabolism. Hence, individuals with increased muscle mass gained from physical activity inherently produce more creatinine. Engaging in regular physical activity can enhance muscle mass,^30^, ^31^ potentially counteracting age‐induced muscle attrition.^32^ On the other hand, a decline in physical activity accentuates the impact of age and other factors on muscle mass reduction. Notably, men tend to undergo a faster reduction in both absolute and relative muscle mass.^33^ Loss of muscle mass subsequently could result in reduced creatinine output, leading to an eGFR_SCr_ increment. This could account for the observed reduced RDKF risk, as characterized by eGFR_SCr_, in individuals who reduced their physical activity during the follow‐up. Such patterns were especially observed in male participants.

Serum CysC levels can vary due to factors such as inflammatory markers, thyroid dysfunction, and adiposity^34^, ^35^; muscle mass is not a factor that influences CysC. Therefore, using CysC to measure eGFR can avoid the confounding effects caused by muscle mass. CysC is generally considered to be sex‐independent.^36^ However, Cystatin C is not without confounders, such as inflammation measured by C‐reactive protein (CRP).^34^, ^35^, ^37^ It is possible that increased physical activity leads to weight loss, reducing resting levels of inflammatory markers and subsequently lowering CRP, which reduces CysC and increases eGFR_CysC_. In the association of changes in PA with eGFR_CysC,_ with baseline and changes in body fat percentage and CRP adjusted for, the effect size of per 1000 MET minutes/week increase in PA was slightly attenuated from 0.04 (95% CI: 0.03, 0.06) to 0.03 (95% CI: 0.01, 0.05), further supporting the suggested mechanisms.

A characteristic of our study population was that average age was below 60 years, and the average kidney function was in the range of 90–95 mL/min/1.73 m^2^, suggesting that they were a cohort with relatively normal kidney function. Additionally, the median physical activity for our study group was around 1900 MET minutes/week, which is considerably above current physical activity guidelines. We believe this is significant because it indicates that even among populations generally considered to be healthy, increasing physical activity is still associated with better kidney health.

Our study demonstrated some sex‐specific differences. When eGFR_SCr_‐defined RDKF was used as the outcome, a decrease in physical activity was protective of RDKF incidence, but only in men. This may be due to the faster decline in both absolute and relative muscle mass in men compared with women, as aforementioned.^33^

As a small exploratory effort, we briefly examined the association between changes in cardiorespiratory fitness and kidney function. Perhaps due to the small sample size, we found almost no meaningful associations. However, we did notice some similarities in the shape of the graphs between this and the primary analysis. It is possible that with a larger sample size, some meaningful findings may be identified.

To the best of our knowledge, no studies have utilized a large sample, along with both eGFR_SCr_ and eGFR_CysC_, to conduct a cohort study of changes in physical activity. One of the major highlights of our study is that we used a large general population study sample to investigate the associations between changes in physical activity and eGFR calculated using different biomarkers. Our findings showed that when measuring the impact of physical activity on kidney function, using CysC yielded more consistent results. With increased physical activity, the incidence of RDKF decreased, and there was an upward trend in the annual change in eGFR. This suggests a potential protective effect of physical activity on kidney function.

However, several points should be noted when interpreting our results. First, physical activity in this study was measured using self‐reported questionnaires rather than objective instruments such as accelerometers. This could introduce a certain degree of self‐reporting bias in the results. Second, although the study suggested a consistent association between changes in physical activity and annual changes in eGFR_CysC_, the effect size was small. Compared with the baseline, an increase of 1000 MET minutes/week in physical activity was associated with an increase of 0.04 mL/min/1.73 m^2^ in eGFR_CysC_. Although statistically significant and consistent across different groups, this may not be large enough to be of clinical significance. In our study population, 2979 participants, or 25.3% of the total, increased their physical activity by 1000 MET minutes/week or more compared with the baseline. The proportion was relatively high. Moreover, an increase of 1000 MET minutes/week was associated with a 4% lower risk of RDKF than those who did not modify their physical activity levels over follow‐up. Therefore, despite small changes at the individual level, there may be significant population‐level benefits. Third, we did not adjust for albuminuria because over 75% of the study participants had urine albumin below the detection threshold. However, all the study participants had available urine creatinine data, meaning the identification of albuminuria could be biased. In addition, albuminuria was regarded as a syndrome of kidney impairment, which was a successor of the study outcome and, therefore, should not be adjusted for. Fourth, GFR was not measured but rather estimated in the UK Biobank. It is plausible that the associations between PA and RDKF were due to non‐GFR effects on biomarkers. Fifth, although we observed a consistent association of total changes in physical activity and RDKF identified by eGFR_SCr_, the upper estimate of the 95% CI was close to null. It meant that while the observed trend suggested a clear association, the statistical significance of this association might not be robust. This indicated the need for cautious interpretation of the data, as the results could be influenced by the narrow confidence intervals. Furthermore, our analysis did not account for some confounders that might increase SCr, such as high‐protein dietary habits commonly found in the UK residents. Without adjusting for these confounders, the SCr was overestimated and the eGFR_SCr_ resulting from the decrease of physical activity was underestimated, thus leading to increased risk of RDKF, showing a marginal association. Nevertheless, our study did not discern an association between changes in physical activity and annual changes in eGFR_SCr_. In addition, a larger sample size might be more powerful in identify tiny differences. Even so, our sample size can detect a 6% decrease of HR with 90% power at a significance level of 0.05, we regarded this sample size as large enough. Last, due to data availability, the changes in eGFR and physical activity were only measured twice. More repeated assessments would offer a better overview of trajectories. This could be addressed in future research should data be available.

In conclusion, the study investigated the complex associations between physical activity levels and kidney function, measured through various biomarkers. Importantly, the study underscored the potentially protective effect of increased physical activity on kidney function, particularly when estimated using eGFR_CysC_. While the study relied on self‐reported physical activity data and showed only modest effect sizes, its large sample size and comprehensive analysis made a compelling case for further research, highlighting the importance of physical activity for chronic disease.

## Conflict of interest

PW reports grant income from Roche Diagnostics, AstraZeneca, Boehringer Ingelheim, and Novartis, outside the submitted work; in addition to consultancy fees from Novo Nordisk and Benecol, outside the current work. PBM reports grant income from AstraZeneca and Boehringer Ingelheim, outside the submitted work; consultancy fees from GSK, Astellas, Bayer, Astra‐Zeneca, Boehringer Ingelheim, outside the current work; in addition to payment from Astra‐Zeneca, Boehringer Ingelheim, Pharmacosmos, outside the current work; in addition to participation on advisory board from Novartis, outside the current work. JSL reports honoraria for lectures from Astra‐Zeneca, outside the current work. NS reports grant income from AstraZeneca, Boehringer Ingelheim, Novartis, and Roche Diagnostics, outside the submitted work; in addition to consulting fees from Abbott Laboratories, Amgen, AstraZeneca, Boehringer Ingelheim, Eli Lilly, Hanmi Pharmaceuticals, Janssen, Merck Sharp & Dohme, Novartis, Novo Nordisk, Pfizer, Roche Diagnostics, and Sanofi, outside the current work; in addition to payment from Abbott Laboratories, AstraZeneca, Boehringer Ingelheim, Eli Lilly, Janssen, and Novo Nordisk, outside the submitted work. Other authors declare no conflict.

## Funding

This research received no specific grant from any funding agency in the public, commercial, or not‐for‐profit sectors.

## Supporting information


**Table S1.** Definition of covariates
**Table S2.** Description of UK Biobank data‐field ID and code value
**Table S3.** Medicine ID and Medicine for Identification of Statin and anti‐cholesterol drugs
**Table S4.** Baseline characteristics of the study population, stratified by the categories of physical activity
**Table S5.** Characteristics of the study population at the follow‐up visit, stratified by the status of RDKF at the end of follow‐up period
**Figure S1.** (a) Association between changes in physical activity and the RDKF incidence identified using eGFR_SCr_ in males. (b) Association between changes in physical activity and the RDKF incidence identified using eGFR_SCr_ in females. (c) Association between changes in physical activity and the RDKF incidence identified using eGFR_SCr_ in people with baseline physical activity above 1,000 MET minutes/week. (d) Association between changes in physical activity and the RDKF incidence identified using eGFR_SCr_ in people with baseline physical activity below 1,000 MET minutes/week.
**Figure S2.** (a) Association between changes in physical activity and the RDKF incidence identified using eGFR_CysC_ in males. (b) Association between changes in physical activity and the RDKF incidence identified using eGFR_CysC_ in females. (c) Association between changes in physical activity and the RDKF incidence identified using eGFR_CysC_ in people with baseline physical activity above 1,000 MET minutes/week. (d) Association between changes in physical activity and the RDKF incidence identified using eGFR_CysC_ in people with baseline physical activity below 1,000 MET minutes/week.
**Figure S3.** (a) Association between changes in physical activity and the RDKF incidence identified using eGFR_SCr‐CysC_. (b) Association between changes in physical activity and the RDKF incidence identified using eGFR_SCr‐CysC_ in males. (c) Association between changes in physical activity and the RDKF incidence identified using eGFR_SCr‐CysC_ in females. (d) Association between changes in physical activity and the RDKF incidence identified using eGFR_SCr‐CysC_ in people with baseline physical activity above 1,000 MET minutes/week. (e) Association between changes in physical activity and the RDKF incidence identified using eGFR_SCr‐CysC_ in people with baseline physical activity below 1,000 MET minutes/week
**Figure S4.** (a) Association between changes in physical activity and the annual change of eGFR_SCr_ in males. (b) Association between changes in physical activity and the annual change of eGFR_SCr_ in females. (c) Association between changes in physical activity and the annual change of eGFR_SCr_ in people with baseline physical activity above 1,000 MET minutes/week. (d) Association between changes in physical activity and the annual change of eGFR_SCr_ in people with baseline physical activity below 1,000 MET minutes/week.
**Figure S5.** (a) Association between changes in physical activity and the annual change of eGFR_CysC_ in males. (b) Association between changes in physical activity and the annual change of eGFR_CysC_ in females. (c) Association between changes in physical activity and the annual change of eGFR_CysC_ in people with baseline physical activity above 1,000 MET minutes/week. (d) Association between changes in physical activity and the annual change of eGFR_CysC_ in people with baseline physical activity below 1,000 MET minutes/week.
**Figure S6.** (a) Association between changes in physical activity and the annual change of eGFR_SCr‐CysC_. (b) Association between changes in physical activity and the annual change of eGFR_SCr‐CysC_ in males. (c) Association between changes in physical activity and the annual change of eGFR_SCr‐CysC_ in females. (d) Association between changes in physical activity and the annual change of eGFR_SCr‐CysC_ in people with baseline physical activity above 1,000 MET minutes/week. (e) Association between changes in physical activity and the annual change of eGFR_SCr‐CysC_ in people with baseline physical activity below 1,000 MET minutes/week.
**Figure S7.** (a) Association between changes in VO_2_max and the RDKF incidence identified using eGFR_SCr_. (b) Association between changes in VO_2_max and the RDKF incidence identified using eGFR_SCr_ in males. (c) Association between changes in VO_2_max and the RDKF incidence identified using eGFR_SCr_ in females.
**Figure S8.** (a) Association between changes in VO_2_max and the RDKF incidence identified using eGFR_CysC_. (b) Association between changes in VO_2_max and the RDKF incidence identified using eGFR_CysC_ in males. (c) Association between changes in VO_2_max and the RDKF incidence identified using eGFR_CysC_ in females.
**Figure S9.** (a) Association between changes in VO_2_max and the annual change of eGFR_SCr_. (b) Association between changes in VO_2_max and the annual change of eGFR_SCr_ in males. (c) Association between changes in VO_2_max and the annual change of eGFR_SCr_ in females.
**Figure S10.** (a) Association between changes in VO_2_max and the annual change of eGFR_CysC_. (b) Association between changes in VO_2_max and the annual change of eGFR_CysC_ in males. (c) Association between changes in VO_2_max and the annual change of eGFR_CysC_ in females

## Data Availability

UK Biobank data are available through application to its official site https://www.ukbiobank.ac.uk/.
