# Supplementary material for: Change in physical activity and its association with decline in kidney function: A UK Biobank‐based cohort study
Source: J Cachexia Sarcopenia Muscle. 2024 Aug 18;15(5):2046–55. doi: 10.1002/jcsm.13551 (PMC11446710; doi:10.1002/jcsm.13551)
Supplement: Supplementary file 1 — Table S1. Definition of covariates Table S2. Description of UK Biobank data‐field ID and code value Table S3. Medicine ID and Medicine for Identification of Statin and anti‐cholesterol drugs Table S4. Baseline characteristics of the study population, stratified by the categories of physical activity Table S5. Characteristics of the study population at the follow‐up visit, stratified by the status of RDKF at the end of follow‐up period Figure S1. (a) Association between changes in physical activity and the RDKF incidence identified using eGFRSCr in males. (b) Association between changes in physical activity and the RDKF incidence identified using eGFRSCr in females. (c) Association between changes in physical activity and the RDKF incidence identified using eGFRSCr in people with baseline physical activity above 1,000 MET minutes/week. (d) Association between changes in physical activity and the RDKF incidence identified using eGFRSCr in people with baseline physical activity below 1,000 MET minutes/week. Figure S2. (a) Association between changes in physical activity and the RDKF incidence identified using eGFRCysC in males. (b) Association between changes in physical activity and the RDKF incidence identified using eGFRCysC in females. (c) Association between changes in physical activity and the RDKF incidence identified using eGFRCysC in people with baseline physical activity above 1,000 MET minutes/week. (d) Association between changes in physical activity and the RDKF incidence identified using eGFRCysC in people with baseline physical activity below 1,000 MET minutes/week. Figure S3. (a) Association between changes in physical activity and the RDKF incidence identified using eGFRSCr‐CysC. (b) Association between changes in physical activity and the RDKF incidence identified using eGFRSCr‐CysC in males. (c) Association between changes in physical activity and the RDKF incidence identified using eGFRSCr‐CysC in females. (d) Association between changes in physical [file JCSM-15-2046-s001.docx]

**Table of Contents**

Supplementary Table 1 Definition of covariates

Supplementary Table 2 Description of UK Biobank data-field ID and code value

Supplementary Table 3 Medicine ID and Medicine for Identification of Statin and anti-cholesterol drugs

Supplementary Table 4 Baseline characteristics of the study population, stratified by the categories of physical activity

Supplementary Table 5 Characteristics of the study population at the follow-up visit, stratified by the status of RDKF at the end of follow-up period

Supplementary Figure 1a Association between changes in physical activity and the RDKF incidence identified using eGFR_SCr_ in males

Supplementary Figure 1b Association between changes in physical activity and the RDKF incidence identified using eGFR_SCr_ in females

Supplementary Figure 1c Association between changes in physical activity and the RDKF incidence identified using eGFR_SCr_ in people with baseline physical activity above 1000 MET minutes/week

Supplementary Figure 1d Association between changes in physical activity and the RDKF incidence identified using eGFR_SCr_ in people with baseline physical activity below 1000 MET minutes/week

Supplementary Figure 2a Association between changes in physical activity and the RDKF incidence identified using eGFR_CysC_ in males

Supplementary Figure 2b Association between changes in physical activity and the RDKF incidence identified using eGFR_CysC_ in females

Supplementary Figure 2c Association between changes in physical activity and the RDKF incidence identified using eGFR_CysC_ in people with baseline physical activity above 1000 MET minutes/week

Supplementary Figure 2d Association between changes in physical activity and the RDKF incidence identified using eGFR_CysC_ in people with baseline physical activity below 1000 MET minutes/week

Supplementary Figure 3a Association between changes in physical activity and the RDKF incidence identified using eGFR_SCr-CysC_

Supplementary Figure 3b Association between changes in physical activity and the RDKF incidence identified using eGFR_SCr-CysC_ in males

Supplementary Figure 3c Association between changes in physical activity and the RDKF incidence identified using eGFR_SCr-CysC_ in females

Supplementary Figure 3d Association between changes in physical activity and the RDKF incidence identified using eGFR_SCr-CysC_ in people with baseline physical activity above 1000 MET minutes/week

Supplementary Figure 3e Association between changes in physical activity and the RDKF incidence identified using eGFR_SCr-CysC_ in people with baseline physical activity below 1000 MET minutes/week

Supplementary Figure 4a Association between changes in physical activity and the annual change of eGFR_SCr_ in males

Supplementary Figure 4b Association between changes in physical activity and the annual change of eGFR_SCr_ in females

Supplementary Figure 4c Association between changes in physical activity and the annual change of eGFR_SCr_ in people with baseline physical activity above 1000 MET minutes/week

Supplementary Figure 4d Association between changes in physical activity and the annual change of eGFR_SCr_ in people with baseline physical activity below 1000 MET minutes/week

Supplementary Figure 5a Association between changes in physical activity and the annual change of eGFR_CysC_ in males

Supplementary Figure 5b Association between changes in physical activity and the annual change of eGFR_CysC_ in females

Supplementary Figure 5c Association between changes in physical activity and the annual change of eGFR_CysC_ in people with baseline physical activity above 1000 MET minutes/week

Supplementary Figure 5d Association between changes in physical activity and the annual change of eGFR_CysC_ in people with baseline physical activity below 1000 MET minutes/week

Supplementary Figure 6a Association between changes in physical activity and the annual change of eGFR_SCr-CysC_

Supplementary Figure 6b Association between changes in physical activity and the annual change of eGFR_SCr-CysC_ in males

Supplementary Figure 6c Association between changes in physical activity and the annual change of eGFR_SCr-CysC_ in females

Supplementary Figure 6d Association between changes in physical activity and the annual change of eGFR_SCr-CysC_ in people with baseline physical activity above 1000 MET minutes/week

Supplementary Figure 6e Association between changes in physical activity and the annual change of eGFR_SCr-CysC_ in people with baseline physical activity below 1000 MET minutes/week

Supplementary Figure 7a Association between changes in VO_2_max and the RDKF incidence identified using eGFR_SCr_

Supplementary Figure 7b Association between changes in VO_2_max and the RDKF incidence identified using eGFR_SCr_ in males

Supplementary Figure 7c Association between changes in VO_2_max and the RDKF incidence identified using eGFR_SCr_ in females

Supplementary Figure 8a Association between changes in VO_2_max and the RDKF incidence identified using eGFR_CysC_

Supplementary Figure 8b Association between changes in VO_2_max and the RDKF incidence identified using eGFR_CysC_ in males

Supplementary Figure 8c Association between changes in VO_2_max and the RDKF incidence identified using eGFR_CysC_ in females

Supplementary Figure 9a Association between changes in VO_2_max and the annual change of eGFR_SCr_

Supplementary Figure 9b Association between changes in VO_2_max and the annual change of eGFR_SCr_ in males

Supplementary Figure 9c Association between changes in VO_2_max and the annual change of eGFR_SCr_ in females

Supplementary Figure 10a Association between changes in VO_2_max and the annual change of eGFR_CysC_

Supplementary Figure 10b Association between changes in VO_2_max and the annual change of eGFR_CysC_ in males

Supplementary Figure 10c Association between changes in VO_2_max and the annual change of eGFR_CysC_ in females

**Supplementary Table 1 Definition of covariates**

| Covariates | Definition |
| --- | --- |
| Sex of the participant | Directly obtained from Data-field ID 31 |
| Race (White, Black, South Asian, and others), | Directly obtained from Data-field ID 21000 |
| Baseline age | Directly obtained from Data-field ID 21003 |
| Baseline systolic and diastolic blood pressures | Blood pressures were calculated as the average of manual reading and automatic reading. For SBP, the Data-field IDs are 4080 (automatic reading) and 93 (manual reading). For DBP, the Data-field IDs are 4079 and 94 respectively. |
| Baseline body mass index | Directly obtained from Data-field ID 23104 |
| Baseline smoking status | Directly obtained from Data-field ID 20116 |
| Self-reported use of statin/anti-cholesterol drugs | Examined Data-field ID 20003, use of statin/anti-cholesterol drugs was identified if the datafield contains any of 1141146234,1141146138,1140888594,1140864592,1140888648,1141192414,1140861958,1140881748,1141157416, 1140861942, 1140865576, 1140861936, 1141172214,1140861858, 1141188546, 1141181868, 1140861884,1140861892, 1140861894, 1140861868,1140861848, 1141192740, 1140861954,1141162544,1141192736,1140862026,  1140888590,1141200040 |
| Self-reported non-cancer illness: |  |
| Hypertension | Examined Data-field ID 20002, this disease was identified if the datafield contains 1065 or 1072, or if baseline SBP over 140 mmHg or DBP over 90 mmHg. |
| Diabetes | Examined Data-field ID 20002, this disease was identified if the datafield contains 1220, 1222, 1223, 1276, 1468, or 1607. Examined Data-field ID 2443, 2976, 5901, and 6177. |
| Chronic heart disease | Examined Data-field ID 20002, chronic heart disease was identified if the datafield contains 1074 or 1075. |
| Chronic obstructive pulmonary disease | Examined Data-field ID 20002, the disease was identified if the datafield contains 1112, 1113, or 1472. |
| Stroke | Examined Data-field ID 20002, the disease was identified if the datafield contains 1081, 1082, 1086, 1491, or 1583 |
| Atrial fibrillation | Examined Data-field ID 20002, the disease was identified if the datafield contains 1471 |
| Heart failure | Examined Data-field ID 20002, the disease was identified if the datafield contains 1076, 1079, or 1588 |
| Myocardial infarction | Examined Data-field ID 20002, the disease was identified if the datafield contains 1075 |
| Townsend deprivation index | Directly obtained from Data-field ID 22189. A higher Townsend index score means a higher degree of deprivation. |

**Supplementary Table 2 Description of UK Biobank data-field ID and code value**

| Data-field ID or Code | Description |
| --- | --- |
| 31 | Sex |
| 93 | Systolic blood pressure, manual reading |
| 94 | Diastolic blood pressure, manual reading |
| 1065 | Hypertension |
| 1072 | Essential hypertension |
| 1074 | Angina |
| 1075 | Heart attack/myocardial infarction |
| 1076 | Heart failure/pulmonary odema |
| 1079 | Cardiomyopathy |
| 1081 | Stroke |
| 1082 | Transient ischaemic attack |
| 1086 | Subarachnoid haemorrhage |
| 1112 | Chronic obstructive airways disease |
| 1113 | Emphysema/chronic bronchitis |
| 1220 | Diabetes |
| 1222 | Type 1 diabetes |
| 1223 | Type 2 diabetes |
| 1276 | Diabetic eye disease |
| 1468 | Diabetic neuropathy/ulcers |
| 1471 | Atrial fibrillation |
| 1472 | Emphysema |
| 1491 | Brain haemorrhage |
| 1583 | Ischaemic stroke |
| 1588 | Hypertrophic cardiomyopathy |
| 1607 | Diabetic nephropathy |
| 2443 | Diabetes diagnosed by doctor |
| 2976 | Age diabetes diagnosed |
| 4079 | Diastolic blood pressure, automated reading |
| 4080 | Systolic blood pressure, automated reading |
| 5901 | Age when diabetes-related eye disease diagnosed |
| 6177 | Medication for cholesterol, blood pressure, or diabetes |
| 20116 | Smoking status |
| 21000 | Ethnic background |
| 21003 | Age when attended assessment center |
| 22189 | Townsend deprivation index at recruitment |
| 23104 | Body mass index |

**Supplementary Table 3 Medicine ID and Medicine for Identification of Statin and anti-cholesterol drugs**

| Medicine ID | Medicine |
| --- | --- |
| 1140861848 | Colestid 5g/sachet granules |
| 1140861858 | Lopid 300 capsule |
| 1140861868 | Nicotinic acid product |
| 1140861884 | Maxepa 1g capsule |
| 1140861892 | Acipimox |
| 1140861894 | Olbetam 250mg capsule |
| 1140861936 | Questran 4g/sachet powder |
| 1140861942 | Cholestyramine+aspartame 4g/sachet powder |
| 1140861954 | Fenofibrate |
| 1140861958 | Simvastatin |
| 1140862026 | Ciprofibrate |
| 1140864592 | Lescol 20mg capsule |
| 1140865576 | Cholestyramine |
| 1140881748 | Zocor 10mg tablet |
| 1140888590 | Colestipol |
| 1140888594 | Fluvastatin |
| 1140888648 | Pravastatin |
| 1141146138 | Lipitor 10mg tablet |
| 1141146234 | Atorvastatin |
| 1141157416 | Cholestyramine product |
| 1141162544 | Lipantil micro 67mg capsule |
| 1141172214 | Supralip 160mg m/r tablet |
| 1141181868 | Omacor 1g capsule |
| 1141188546 | Niaspan 500mg m/r tablet |
| 1141192414 | Crestor 10mg tablet |
| 1141192736 | Ezetimibe |
| 1141192740 | Ezetrol 10mg tablet |
| 1141200040 | Zocor heart-pro 10mg tablet |

**Supplementary Table 4 Baseline characteristics of the study population, stratified by the categories of physical activity**

|  | All | Baseline PA< 1000 MET minutes/week | Baseline PA≥1000 MET minutes/week |
| --- | --- | --- | --- |
| Sample size, n (%)^a^ | 11757 (100) | 3285 (27.94) | 8472 (72.06) |
| Age, years | 57.25 (7.35) | 56.79 (7.34) | 57.43 (7.35) |
| Male, n (%) | 5978 (50.85) | 1559 (47.46) | 4419 (52.16) |
| Ethnicity, n (%) |  |  |  |
| White | 11530 (98.07) | 3217 (97.93) | 8313 (98.12) |
| Black | 47 (0.40) | 15 (0.46) | 32 (0.38) |
| South Asian | 61 (0.52) | 22 (0.67) | 39 (0.46) |
| Others | 119 (1.01) | 31 (0.94) | 88 (1.04) |
| Body mass index, kg/m^2^ | 26.46 (4.13) | 26.93 (4.45) | 26.28 (3.98) |
| Smoking, n (%) |  |  |  |
| Never | 7030 (59.79) | 1979 (60.24) | 5051 (59.62) |
| Previous | 4026 (34.24) | 1111 (33.82) | 2915 (34.41) |
| Current | 701 (5.96) | 195 (5.94) | 506 (5.97) |
| Townsend deprivation index, median (IQR) | -2.76 (3.13) | -2.78 (3.13) | -2.76 (3.13) |
| Systolic blood pressure, mmHg | 137.72 (18.15) | 136.77 (18.03) | 138.09 (18.18) |
| Diastolic blood pressure, mmHg | 81.52 (9.79) | 81.58 (9.82) | 81.50 (9.79) |
| Serum creatinine, mg/dL | 0.81 (0.15) | 0.81 (0.15) | 0.81 (0.15) |
| Serum cystatin C, mg/L | 0.87 (0.11) | 0.88 (0.11) | 0.87 (0.11) |
| C-reactive protein, mg/L, median (IQR) | 1.08 (1.64) | 1.24 (1.82) | 1.03 (1.56) |
| Estimated glomerular filtration rate, mean (SD) |  |  |  |
| eGFR_SCr_, ml/min/1.73m^2^ | 95.44 (10.90) | 95.62 (10.94) | 95.36 (10.88) |
| eGFR_CysC_, ml/min/1.73m^2^ | 91.28 (13.24) | 90.82 (13.31) | 91.45 (13.21) |
| Comorbidities, n (%) |  |  |  |
| Atrial fibrillation | 79 (0.67) | 21 (0.64) | 58 (0.68) |
| Chronic obstructive pulmonary disease | 99 (0.84) | 20 (0.61) | 79 (0.93) |
| Coronary heart disease | 365 (3.10) | 94 (2.86) | 271 (3.20) |
| Diabetes | 405 (3.44) | 125 (3.81) | 280 (3.31) |
| Heart failure | 6 (0.05) | 1 (0.03) | 5 (0.06) |
| Hypertension | 5867 (49.90) | 1650 (50.23) | 4217 (49.78) |
| Myocardial infarction | 199 (1.69) | 53 (1.61) | 146 (1.72) |
| Stroke | 156 (1.33) | 51 (1.55) | 105 (1.24) |
| Use of statin, n (%) | 1632 (13.88) | 472 (14.37) | 1160 (13.69) |
| Total MET minutes/week of physical activity, median (IQR) | 1896 (2723) | 594 (348) | 2706 (2944) |
| VO_2_max, ml/kg/min, median (IQR)^b^ | 38.19 (73.87) | 36.64 (78.33) | 38.65 (72.88) |

CysC Cystatin C, eGFR estimated glomerular filtration rate, IQR Interquartile range, MET Metabolic equivalent task, PA physical activity, RDKF Rapid decline of kidney function, SCr Serum creatinine, SD standard deviation

% is the column percentage unless otherwise specified

^a^row percentage is presented

^b^sample size=2,040 participants

**Supplementary Table 5 Characteristics of the study population at the follow-up visit, stratified by the status of RDKF at the end of follow-up period**

|  | All |  | RDKF by eGFR_SCr_ | |  | RDKF by eGFR_CysC_ | |
| --- | --- | --- | --- | --- | --- | --- | --- |
|  |  |  | Yes | No |  | Yes | No |
| Sample size, n (%)^a^ | 11757 (100) |  | 1731 (14.72) | 10026 (85.28) |  | 1285 (10.93) | 10472 (89.07) |
| Age, years | 61.53 (7.34) |  | 61.38 (7.35) | 61.56 (7.33) |  | 62.82 (7.01) | 61.38 (7.36) |
| Male, n (%) | 5978 (50.85) |  | 869 (50.20) | 5109 (50.96) |  | 744 (57.90) | 5234 (49.98) |
| Ethnicity, n (%) |  |  |  |  |  |  |  |
| White | 11530 (98.07) |  | 1701 (98.27) | 9829 (98.04) |  | 1259 (97.98) | 10271 (98.08) |
| Black | 47 (0.40) |  | 6 (0.35) | 41 (0.41) |  | 3 (0.23) | 44 (0.42) |
| South Asian | 61 (0.52) |  | 6 (0.35) | 55 (0.55) |  | 9 (0.70) | 52 (0.50) |
| Others | 119 (1.01) |  | 18 (1.04) | 101 (1.01) |  | 14 (1.09) | 105 (1.00) |
| Body mass index, kg/m^2^ | 26.54 (4.19) |  | 26.99 (4.25) | 26.46 (4.18) |  | 27.54 (4.72) | 26.42 (4.11) |
| Smoking, n (%) |  |  |  |  |  |  |  |
| Never | 7133 (60.67) |  | 1048 (60.54) | 6085 (60.69) |  | 740 (57.59) | 6393 (61.05) |
| Previous | 4142 (35.23) |  | 616 (35.59) | 3526 (35.17) |  | 471 (36.65) | 3671 (35.06) |
| Current | 482 (4.10) |  | 67 (3.87) | 415 (4.14) |  | 74 (5.76) | 408 (3.90) |
| Townsend deprivation index, median (IQR) | -2.76 (3.13) |  | -2.83 (3.18) | -2.76 (3.12) |  | -2.69 (3.12) | -2.77 (3.12) |
| Systolic blood pressure, mmHg | 139.15 (18.37) |  | 138.46 (18.28) | 139.27 (18.38) |  | 140.15 (18.80) | 139.02 (18.31) |
| Diastolic blood pressure, mmHg | 80.25 (9.65) |  | 79.96 (9.71) | 80.30 (9.63) |  | 80.78 (10.44) | 80.19 (9.54) |
| Serum creatinine, mg/dL | 0.84 (0.16) |  | 0.99 (0.17) | 0.81 (0.15) |  | 0.91 (0.18) | 0.83 (0.16) |
| Serum cystatin C, mg/L | 0.90 (0.13) |  | 0.94 (0.16) | 0.89 (0.13) |  | 1.03 (0.14) | 0.88 (0.12) |
| C-reactive protein, mg/L, median (IQR) | 1.12 (1.67) |  | 1.22 (1.73) | 1.11 (1.65) |  | 1.64 (2.27) | 1.06 (1.61) |
| Estimated glomerular filtration rate, mean (SD) |  |  |  |  |  |  |  |
| eGFR_SCr_, ml/min/1.73m^2^ | 90.67 (11.90) |  | 77.35 (11.12) | 92.97 (10.43) |  | 85.16 (13.47) | 91.35 (11.52) |
| eGFR_CysC_, ml/min/1.73m^2^ | 81.71 (14.86) |  | 83.54 (16.12) | 88.43 (14.51) |  | 73.78 (12.69) | 89.42 (14.19) |
| eGFR annual change, median (IQR) |  |  |  |  |  |  |  |
| eGFR_SCr_, ml/min/1.73m^2^ | -0.92 (1.90) |  | -4.19 (1.76) | -0.72 (1.46) |  | -1.68 (2.75) | -0.86 (1.78) |
| eGFR_CysC_, ml/min/1.73m^2^ | -0.88 (1.75) |  | -1.42 (2.40) | -0.80 (2.05) |  | -3.81 (1.29) | -0.68 (1.19) |
| Comorbidities, n (%) |  |  |  |  |  |  |  |
| Atrial fibrillation | 167 (1.42) |  | 33 (1.91) | 134 (1.34) |  | 34 (2.65) | 133 (1.27) |
| Chronic obstructive pulmonary disease | 132 (1.12) |  | 21 (1.21) | 111 (1.11) |  | 15 (1.17) | 117 (1.12) |
| Coronary heart disease | 484 (4.12) |  | 84 (4.85) | 400 (3.99) |  | 77 (5.99) | 407 (3.89) |
| Diabetes | 547 (4.65) |  | 99 (5.72) | 448 (4.47) |  | 99 (7.70) | 448 (4.28) |
| Heart failure | 15 (0.13) |  | 5 (0.29) | 10 (0.10) |  | 4 (0.31) | 11 (0.11) |
| Hypertension | 6642 (56.49) |  | 996 (57.54) | 5646 (56.31) |  | 813 (63.27) | 5829 (55.66) |
| Myocardial infarction | 257 (2.19) |  | 48 (2.77) | 209 (2.08) |  | 39 (3.04) | 218 (2.08) |
| Stroke | 236 (2.01) |  | 30 (1.73) | 206 (2.05) |  | 22 (1.71) | 214 (2.04) |
| Use of statin, n (%) | 2432 (20.69) |  | 392 (22.65) | 2040 (20.35) |  | 343 (26.69) | 2089 (19.95) |
| Total MET minutes/week of physical activity, median (IQR) | 1912 (2604) |  | 1890 (2622) | 1920 (2613) |  | 1800 (2507) | 1920 (2621) |
| VO_2_max, ml/kg/min, median (IQR)^b^ | 37.71 (83.32) |  | 37.94 (90.40) | 37.59 (81.02) |  | 36.06 (80.40) | 37.86 (84.62) |

CysC Cystatin C, eGFR estimated glomerular filtration rate, IQR Interquartile range, MET Metabolic equivalent task, RDKF Rapid decline of kidney function,

SCr Serum creatinine, SD standard deviation

% is the column percentage unless otherwise specified

^a^row percentage is presented

^b^sample size=2,040 participants

**Supplementary Figure 1a Association between changes in physical activity and the RDKF incidence identified using eGFR_SCr_ in males**

The solid black line represents the regression line. Dashed lines on either side of the solid black line show the 95% confidence interval (CI). The red line is for easy reference, and a 95%CI below or above the line is regarded as a meaningful association.

Adjusted for race, baseline age, smoking, body mass index, Townsend deprivation index, baseline systolic blood pressure, baseline diastolic pressure, use of statin, hypertension, diabetes, coronary heart disease, chronic obstructive pulmonary disease, stroke, atrial fibrillation, heart failure, myocardial infarction.

**Supplementary Figure 1b Association between changes in physical activity and the RDKF incidence identified using eGFR_SCr_ in females**

The solid black line represents the regression line. Dashed lines on either side of the solid black line show the 95% confidence interval (CI). The red line is for easy reference, and a 95%CI below or above the line is regarded as a meaningful association.

Adjusted for race, baseline age, smoking, body mass index, Townsend deprivation index, baseline systolic blood pressure, baseline diastolic pressure, use of statin, hypertension, diabetes, coronary heart disease, chronic obstructive pulmonary disease, stroke, atrial fibrillation, heart failure, myocardial infarction.

**Supplementary Figure 1c Association between changes in physical activity and the RDKF incidence identified using eGFR_SCr_ in people with baseline physical activity above 1000 MET minutes/week**

The solid black line represents the regression line. Dashed lines on either side of the solid black line show the 95% confidence interval (CI). The red line is for easy reference, and a 95%CI below or above the line is regarded as a meaningful association.

Adjusted for sex, race, baseline age, smoking, body mass index, Townsend deprivation index, baseline systolic blood pressure, baseline diastolic pressure, use of statin, hypertension, diabetes, coronary heart disease, chronic obstructive pulmonary disease, stroke, atrial fibrillation, heart failure, myocardial infarction.

**Supplementary Figure 1d Association between changes in physical activity and the RDKF incidence identified using eGFR_SCr_ in people with baseline physical activity below 1000 MET minutes/week**

The solid black line represents the regression line. Dashed lines on either side of the solid black line show the 95% confidence interval (CI). The red line is for easy reference, and a 95%CI below or above the line is regarded as a meaningful association.

Adjusted for sex, race, baseline age, smoking, body mass index, Townsend deprivation index, baseline systolic blood pressure, baseline diastolic pressure, use of statin, hypertension, diabetes, coronary heart disease, chronic obstructive pulmonary disease, stroke, atrial fibrillation, heart failure, myocardial infarction.

**Supplementary Figure 2a Association between changes in physical activity and the RDKF incidence identified using eGFR_CysC_ in males**

The solid black line represents the regression line. Dashed lines on either side of the solid black line show the 95% confidence interval (CI). The red line is for easy reference, and a 95%CI below or above the line is regarded as a meaningful association.

Adjusted for race, baseline age, smoking, body mass index, Townsend deprivation index, baseline systolic blood pressure, baseline diastolic pressure, use of statin, hypertension, diabetes, coronary heart disease, chronic obstructive pulmonary disease, stroke, atrial fibrillation, heart failure, myocardial infarction.

**Supplementary Figure 2b Association between changes in physical activity and the RDKF incidence identified using eGFR_CysC_ in females**

The solid black line represents the regression line. Dashed lines on either side of the solid black line show the 95% confidence interval (CI). The red line is for easy reference, and a 95%CI below or above the line is regarded as a meaningful association.

Adjusted for race, baseline age, smoking, body mass index, Townsend deprivation index, baseline systolic blood pressure, baseline diastolic pressure, use of statin, hypertension, diabetes, coronary heart disease, chronic obstructive pulmonary disease, stroke, atrial fibrillation, heart failure, myocardial infarction.

**Supplementary Figure 2c Association between changes in physical activity and the RDKF incidence identified using eGFR_CysC_ in people with baseline physical activity above 1000 MET minutes/week**

The solid black line represents the regression line. Dashed lines on either side of the solid black line show the 95% confidence interval (CI). The red line is for easy reference, and a 95%CI below or above the line is regarded as a meaningful association.

Adjusted for sex, race, baseline age, smoking, body mass index, Townsend deprivation index, baseline systolic blood pressure, baseline diastolic pressure, use of statin, hypertension, diabetes, coronary heart disease, chronic obstructive pulmonary disease, stroke, atrial fibrillation, heart failure, myocardial infarction.

**Supplementary Figure 2d Association between changes in physical activity and the RDKF incidence identified using eGFR_CysC_ in people with baseline physical activity below 1000 MET minutes/week**

The solid black line represents the regression line. Dashed lines on either side of the solid black line show the 95% confidence interval (CI). The red line is for easy reference, and a 95%CI below or above the line is regarded as a meaningful association.

Adjusted for sex, race, baseline age, smoking, body mass index, Townsend deprivation index, baseline systolic blood pressure, baseline diastolic pressure, use of statin, hypertension, diabetes, coronary heart disease, chronic obstructive pulmonary disease, stroke, atrial fibrillation, heart failure, myocardial infarction.

**Supplementary Figure 3a Association between changes in physical activity and the RDKF incidence identified using eGFR_SCr-CysC_**

The solid black line represents the regression line. Dashed lines on either side of the solid black line show the 95% confidence interval (CI). The red line is for easy reference, and a 95%CI below or above the line is regarded as a meaningful association.

Adjusted for sex, race, baseline age, smoking, body mass index, Townsend deprivation index, baseline systolic blood pressure, baseline diastolic pressure, use of statin, hypertension, diabetes, coronary heart disease, chronic obstructive pulmonary disease, stroke, atrial fibrillation, heart failure, myocardial infarction.

**Supplementary Figure 3b Association between changes in physical activity and the RDKF incidence identified using eGFR_SCr-CysC_ in males**

The solid black line represents the regression line. Dashed lines on either side of the solid black line show the 95% confidence interval (CI). The red line is for easy reference, and a 95%CI below or above the line is regarded as a meaningful association.

Adjusted for race, baseline age, smoking, body mass index, Townsend deprivation index, baseline systolic blood pressure, baseline diastolic pressure, use of statin, hypertension, diabetes, coronary heart disease, chronic obstructive pulmonary disease, stroke, atrial fibrillation, heart failure, myocardial infarction.

**Supplementary Figure 3c Association between changes in physical activity and the RDKF incidence identified using eGFR_SCr-CysC_ in females**

The solid black line represents the regression line. Dashed lines on either side of the solid black line show the 95% confidence interval (CI). The red line is for easy reference, and a 95%CI below or above the line is regarded as a meaningful association.

Adjusted for race, baseline age, smoking, body mass index, Townsend deprivation index, baseline systolic blood pressure, baseline diastolic pressure, use of statin, hypertension, diabetes, coronary heart disease, chronic obstructive pulmonary disease, stroke, atrial fibrillation, heart failure, myocardial infarction.

**Supplementary Figure 3d Association between changes in physical activity and the RDKF incidence identified using eGFR_SCr-CysC_ in people with baseline physical activity above 1000 MET minutes/week**

The solid black line represents the regression line. Dashed lines on either side of the solid black line show the 95% confidence interval (CI). The red line is for easy reference, and a 95%CI below or above the line is regarded as a meaningful association.

Adjusted for sex, race, baseline age, smoking, body mass index, Townsend deprivation index, baseline systolic blood pressure, baseline diastolic pressure, use of statin, hypertension, diabetes, coronary heart disease, chronic obstructive pulmonary disease, stroke, atrial fibrillation, heart failure, myocardial infarction.

**Supplementary Figure 3e Association between changes in physical activity and the RDKF incidence identified using eGFR_SCr-CysC_ in people with baseline physical activity below 1000 MET minutes/week**

The solid black line represents the regression line. Dashed lines on either side of the solid black line show the 95% confidence interval (CI). The red line is for easy reference, and a 95%CI below or above the line is regarded as a meaningful association.

Adjusted for sex, race, baseline age, smoking, body mass index, Townsend deprivation index, baseline systolic blood pressure, baseline diastolic pressure, use of statin, hypertension, diabetes, coronary heart disease, chronic obstructive pulmonary disease, stroke, atrial fibrillation, heart failure, myocardial infarction.

**Supplementary Figure 4a Association between changes in physical activity and the annual change of eGFR_SCr_ in males**

The solid black line represents the regression line. Dashed lines on either side of the solid black line show the 95% confidence interval (CI). The red line is for easy reference, and a 95%CI below or above the line is regarded as a meaningful association.

Adjusted for race, baseline age, smoking, body mass index, Townsend deprivation index, baseline systolic blood pressure, baseline diastolic pressure, use of statin, hypertension, diabetes, coronary heart disease, chronic obstructive pulmonary disease, stroke, atrial fibrillation, heart failure, myocardial infarction.

**Supplementary Figure 4b Association between changes in physical activity and the annual change of eGFR_SCr_ in females**

The solid black line represents the regression line. Dashed lines on either side of the solid black line show the 95% confidence interval (CI). The red line is for easy reference, and a 95%CI below or above the line is regarded as a meaningful association.

Adjusted for race, baseline age, smoking, body mass index, Townsend deprivation index, baseline systolic blood pressure, baseline diastolic pressure, use of statin, hypertension, diabetes, coronary heart disease, chronic obstructive pulmonary disease, stroke, atrial fibrillation, heart failure, myocardial infarction.

**Supplementary Figure 4c Association between changes in physical activity and the annual change of eGFR_SCr_ in people with baseline physical activity above 1000 MET minutes/week**

The solid black line represents the regression line. Dashed lines on either side of the solid black line show the 95% confidence interval (CI). The red line is for easy reference, and a 95%CI below or above the line is regarded as a meaningful association.

Adjusted for sex, race, baseline age, smoking, body mass index, Townsend deprivation index, baseline systolic blood pressure, baseline diastolic pressure, use of statin, hypertension, diabetes, coronary heart disease, chronic obstructive pulmonary disease, stroke, atrial fibrillation, heart failure, myocardial infarction.

**Supplementary Figure 4d Association between changes in physical activity and the annual change of eGFR_SCr_ in people with baseline physical activity below 1000 MET minutes/week**

The solid black line represents the regression line. Dashed lines on either side of the solid black line show the 95% confidence interval (CI). The red line is for easy reference, and a 95%CI below or above the line is regarded as a meaningful association.

Adjusted for sex, race, baseline age, smoking, body mass index, Townsend deprivation index, baseline systolic blood pressure, baseline diastolic pressure, use of statin, hypertension, diabetes, coronary heart disease, chronic obstructive pulmonary disease, stroke, atrial fibrillation, heart failure, myocardial infarction.

**Supplementary Figure 5a Association between changes in physical activity and the annual change of eGFR_CysC_ in males**

The solid black line represents the regression line. Dashed lines on either side of the solid black line show the 95% confidence interval (CI). The red line is for easy reference, and a 95%CI below or above the line is regarded as a meaningful association.

Adjusted for race, baseline age, smoking, body mass index, Townsend deprivation index, baseline systolic blood pressure, baseline diastolic pressure, use of statin, hypertension, diabetes, coronary heart disease, chronic obstructive pulmonary disease, stroke, atrial fibrillation, heart failure, myocardial infarction.

**Supplementary Figure 5b Association between changes in physical activity and the annual change of eGFR_CysC_ in females**

The solid black line represents the regression line. Dashed lines on either side of the solid black line show the 95% confidence interval (CI). The red line is for easy reference, and a 95%CI below or above the line is regarded as a meaningful association.

Adjusted for race, baseline age, smoking, body mass index, Townsend deprivation index, baseline systolic blood pressure, baseline diastolic pressure, use of statin, hypertension, diabetes, coronary heart disease, chronic obstructive pulmonary disease, stroke, atrial fibrillation, heart failure, myocardial infarction.

**Supplementary Figure 5c Association between changes in physical activity and the annual change of eGFR_CysC_ in people with baseline physical activity above 1000 MET minutes/week**

The solid black line represents the regression line. Dashed lines on either side of the solid black line show the 95% confidence interval (CI). The red line is for easy reference, and a 95%CI below or above the line is regarded as a meaningful association.

Adjusted for sex, race, baseline age, smoking, body mass index, Townsend deprivation index, baseline systolic blood pressure, baseline diastolic pressure, use of statin, hypertension, diabetes, coronary heart disease, chronic obstructive pulmonary disease, stroke, atrial fibrillation, heart failure, myocardial infarction.

**Supplementary Figure 5d Association between changes in physical activity and the annual change of eGFR_CysC_ in people with baseline physical activity below 1000 MET minutes/week**

The solid black line represents the regression line. Dashed lines on either side of the solid black line show the 95% confidence interval (CI). The red line is for easy reference, and a 95%CI below or above the line is regarded as a meaningful association.

Adjusted for sex, race, baseline age, smoking, body mass index, Townsend deprivation index, baseline systolic blood pressure, baseline diastolic pressure, use of statin, hypertension, diabetes, coronary heart disease, chronic obstructive pulmonary disease, stroke, atrial fibrillation, heart failure, myocardial infarction.

**Supplementary Figure 6a Association between changes in physical activity and the annual change of eGFR_SCr-CysC_**

The solid black line represents the regression line. Dashed lines on either side of the solid black line show the 95% confidence interval (CI). The red line is for easy reference, and a 95%CI below or above the line is regarded as a meaningful association.

Adjusted for sex, race, baseline age, smoking, body mass index, Townsend deprivation index, baseline systolic blood pressure, baseline diastolic pressure, use of statin, hypertension, diabetes, coronary heart disease, chronic obstructive pulmonary disease, stroke, atrial fibrillation, heart failure, myocardial infarction.

**Supplementary Figure 6b Association between changes in physical activity and the annual change of eGFR_SCr-CysC_ in males**

The solid black line represents the regression line. Dashed lines on either side of the solid black line show the 95% confidence interval (CI). The red line is for easy reference, and a 95%CI below or above the line is regarded as a meaningful association.

Adjusted for race, baseline age, smoking, body mass index, Townsend deprivation index, baseline systolic blood pressure, baseline diastolic pressure, use of statin, hypertension, diabetes, coronary heart disease, chronic obstructive pulmonary disease, stroke, atrial fibrillation, heart failure, myocardial infarction.

**Supplementary Figure 6c Association between changes in physical activity and the annual change of eGFR_SCr-CysC_ in females**

The solid black line represents the regression line. Dashed lines on either side of the solid black line show the 95% confidence interval (CI). The red line is for easy reference, and a 95%CI below or above the line is regarded as a meaningful association.

Adjusted for race, baseline age, smoking, body mass index, Townsend deprivation index, baseline systolic blood pressure, baseline diastolic pressure, use of statin, hypertension, diabetes, coronary heart disease, chronic obstructive pulmonary disease, stroke, atrial fibrillation, heart failure, myocardial infarction.

**Supplementary Figure 6d Association between changes in physical activity and the annual change of eGFR_SCr-CysC_ in people with baseline physical activity above 1000 MET minutes/week**

The solid black line represents the regression line. Dashed lines on either side of the solid black line show the 95% confidence interval (CI). The red line is for easy reference, and a 95%CI below or above the line is regarded as a meaningful association.

Adjusted for sex, race, baseline age, smoking, body mass index, Townsend deprivation index, baseline systolic blood pressure, baseline diastolic pressure, use of statin, hypertension, diabetes, coronary heart disease, chronic obstructive pulmonary disease, stroke, atrial fibrillation, heart failure, myocardial infarction.

**Supplementary Figure 6e Association between changes in physical activity and the annual change of eGFR_SCr-CysC_ in people with baseline physical activity below 1000 MET minutes/week**

The solid black line represents the regression line. Dashed lines on either side of the solid black line show the 95% confidence interval (CI). The red line is for easy reference, and a 95%CI below or above the line is regarded as a meaningful association.

Adjusted for sex, race, baseline age, smoking, body mass index, Townsend deprivation index, baseline systolic blood pressure, baseline diastolic pressure, use of statin, hypertension, diabetes, coronary heart disease, chronic obstructive pulmonary disease, stroke, atrial fibrillation, heart failure, myocardial infarction.

**Supplementary Figure 7a Association between changes in VO_2_max and the RDKF incidence identified using eGFR_SCr_**


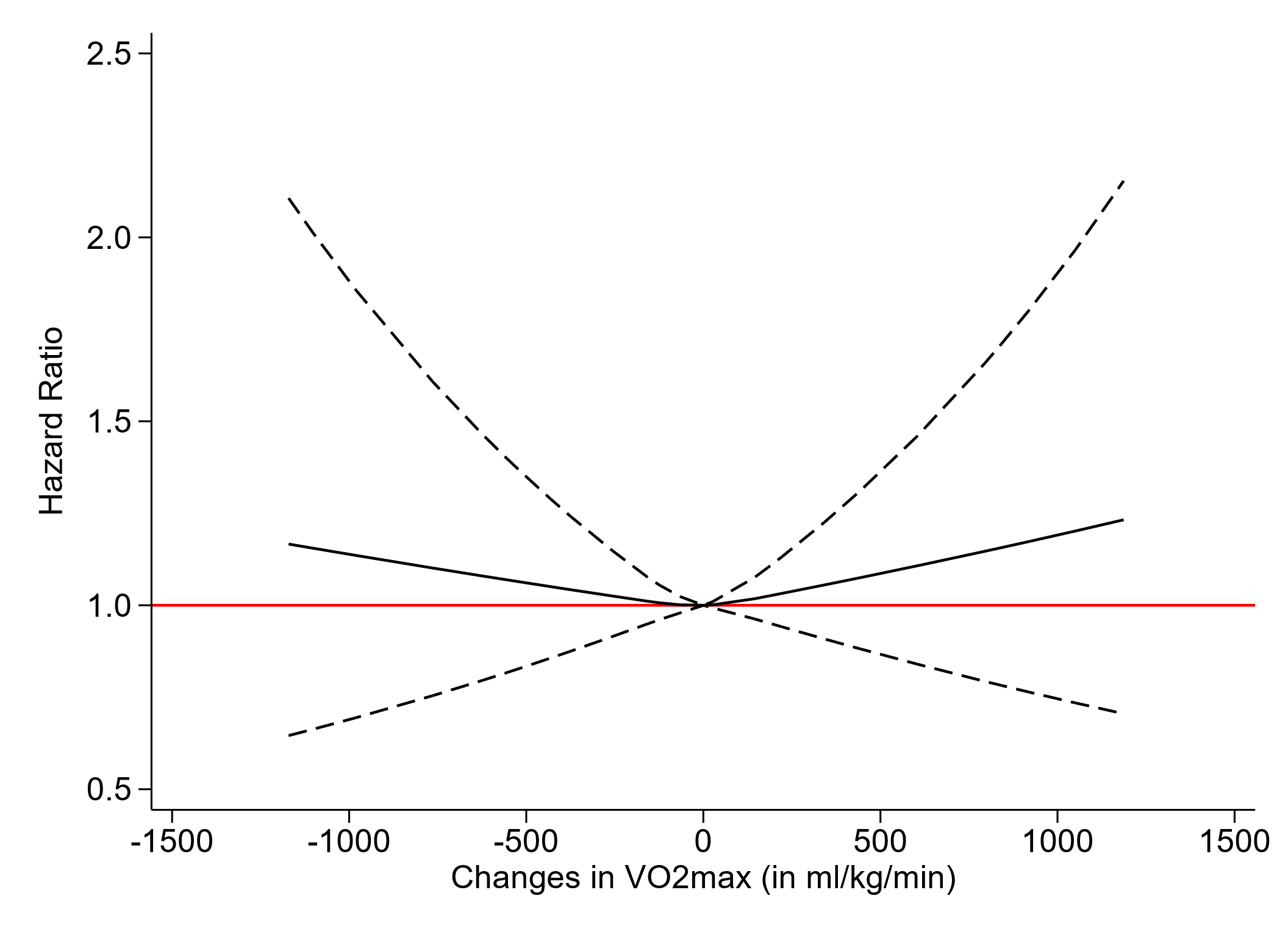


The solid black line represents the regression line. Dashed lines on either side of the solid black line show the 95% confidence interval (CI). The red line is for easy reference, and a 95%CI below or above the line is regarded as a meaningful association.

Adjusted for sex, race, baseline age, smoking, body mass index, Townsend deprivation index, baseline systolic blood pressure, baseline diastolic pressure, use of statin, hypertension, diabetes, coronary heart disease, chronic obstructive pulmonary disease, stroke, atrial fibrillation, heart failure, myocardial infarction.

**Supplementary Figure 7b Association between changes in VO_2_max and the RDKF incidence identified using eGFR_SCr_ in males**


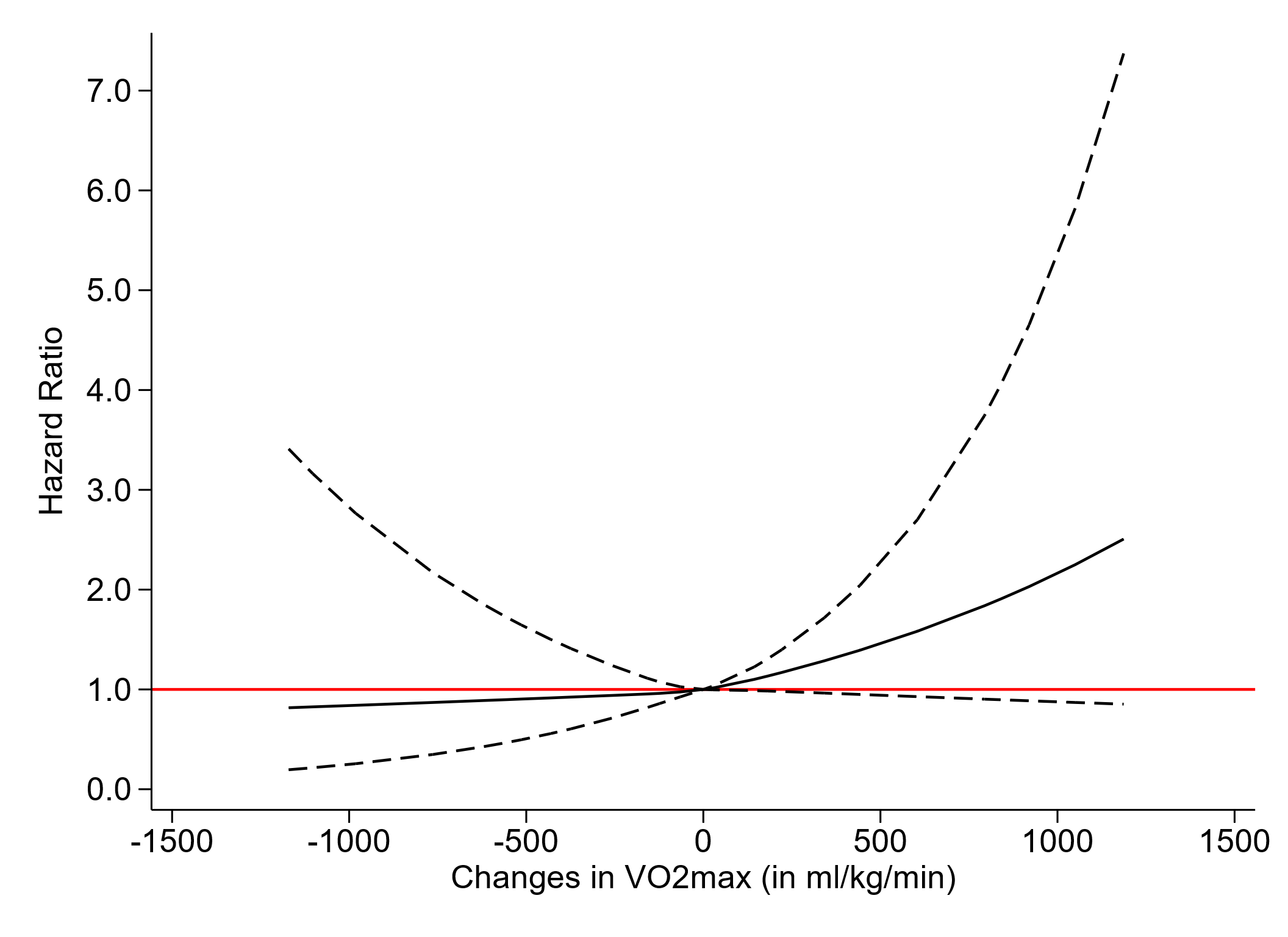


The solid black line represents the regression line. Dashed lines on either side of the solid black line show the 95% confidence interval (CI). The red line is for easy reference, and a 95%CI below or above the line is regarded as a meaningful association.

Adjusted for race, baseline age, smoking, body mass index, Townsend deprivation index, baseline systolic blood pressure, baseline diastolic pressure, use of statin, hypertension, diabetes, coronary heart disease, chronic obstructive pulmonary disease, stroke, atrial fibrillation, heart failure, myocardial infarction.

**Supplementary Figure 7c Association between changes in VO_2_max and the RDKF incidence identified using eGFR_SCr_ in females**


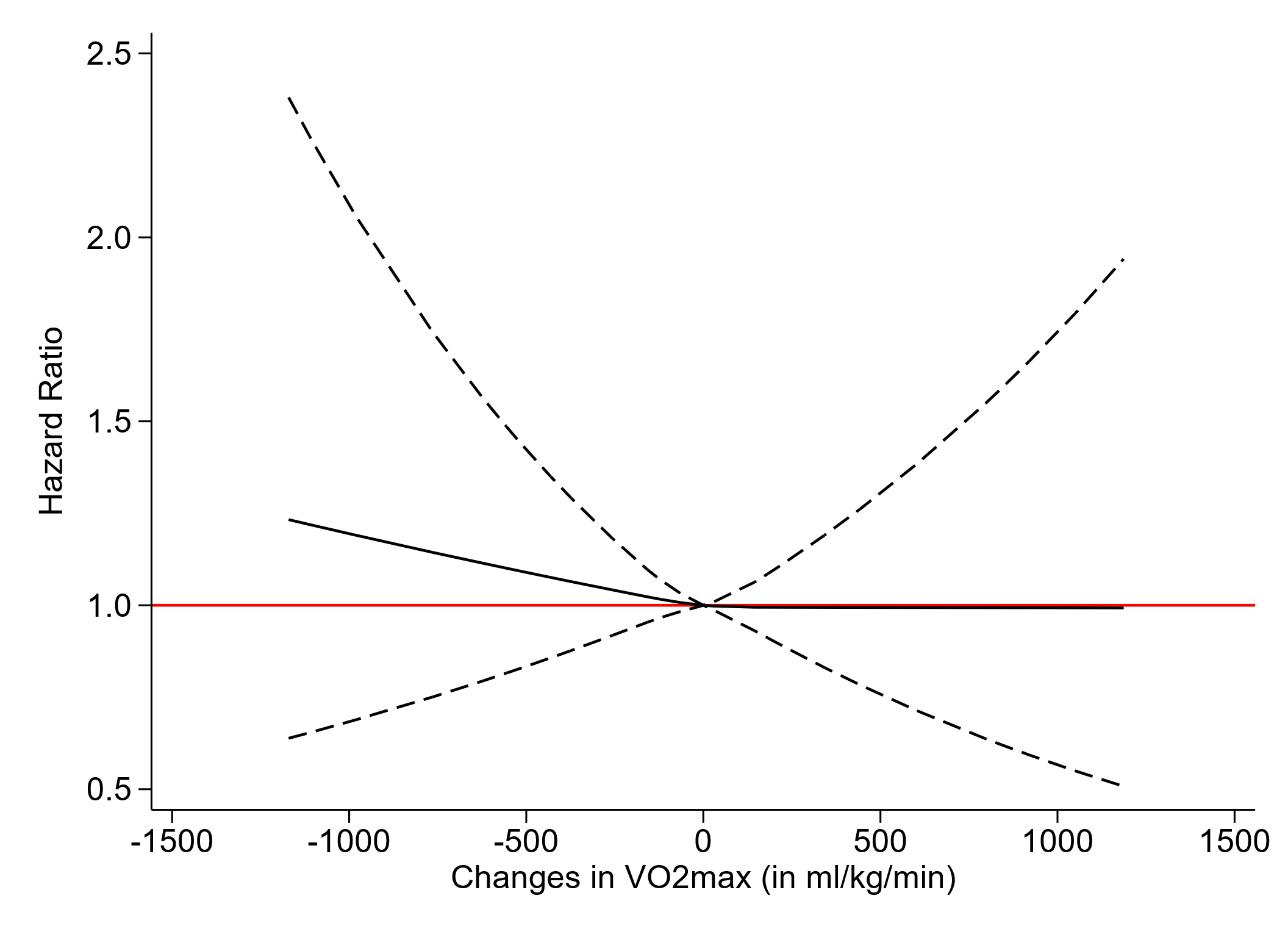


The solid black line represents the regression line. Dashed lines on either side of the solid black line show the 95% confidence interval (CI). The red line is for easy reference, and a 95%CI below or above the line is regarded as a meaningful association.

Adjusted for race, baseline age, smoking, body mass index, Townsend deprivation index, baseline systolic blood pressure, baseline diastolic pressure, use of statin, hypertension, diabetes, coronary heart disease, chronic obstructive pulmonary disease, stroke, atrial fibrillation, heart failure, myocardial infarction.

**Supplementary Figure 8a Association between changes in VO_2_max and the RDKF incidence identified using eGFR_CysC_**


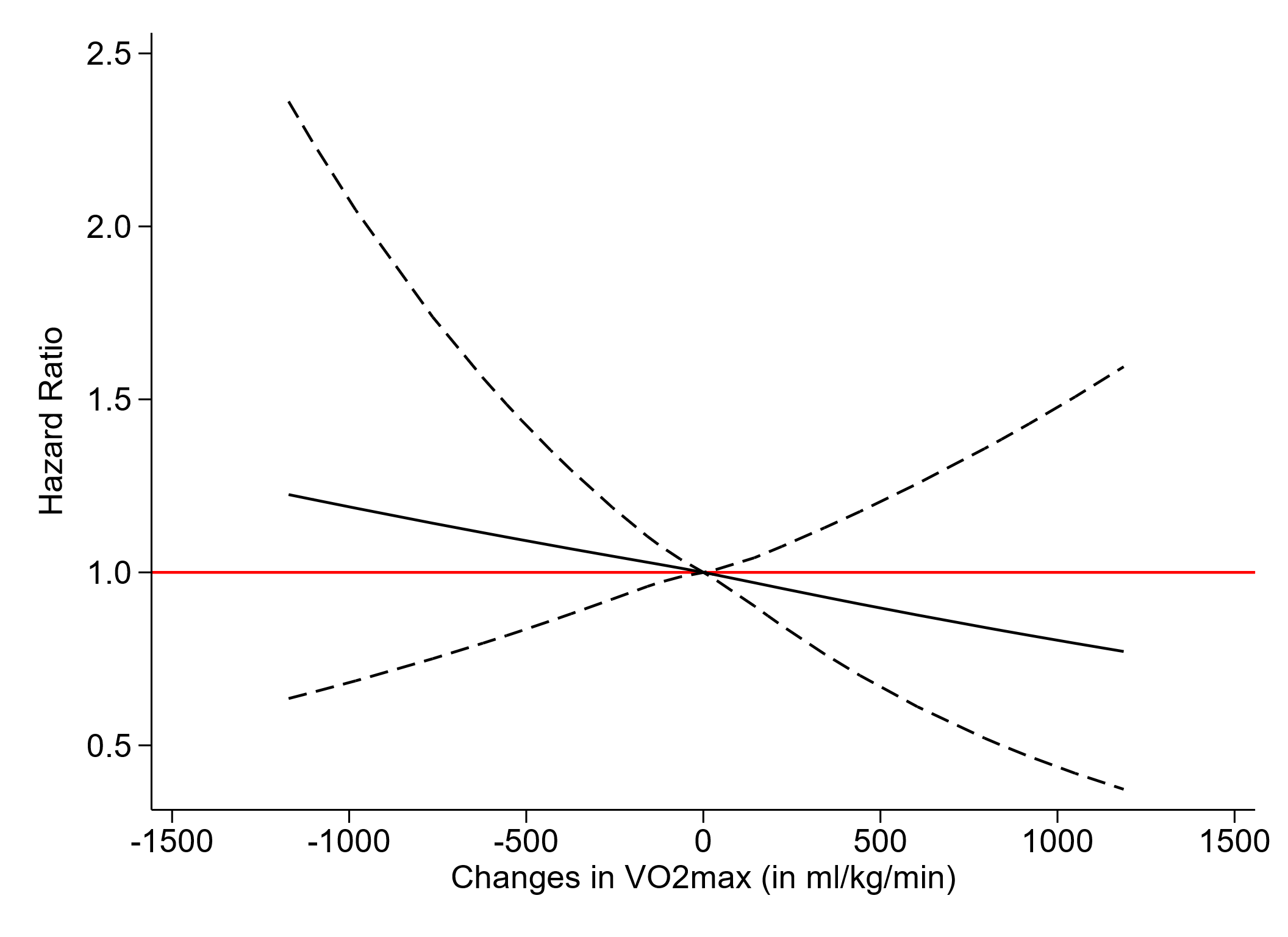


The solid black line represents the regression line. Dashed lines on either side of the solid black line show the 95% confidence interval (CI). The red line is for easy reference, and a 95%CI below or above the line is regarded as a meaningful association.

Adjusted for sex, race, baseline age, smoking, body mass index, Townsend deprivation index, baseline systolic blood pressure, baseline diastolic pressure, use of statin, hypertension, diabetes, coronary heart disease, chronic obstructive pulmonary disease, stroke, atrial fibrillation, heart failure, myocardial infarction.

**Supplementary Figure 8b Association between changes in VO_2_max and the RDKF incidence identified using eGFR_CysC_ in males**


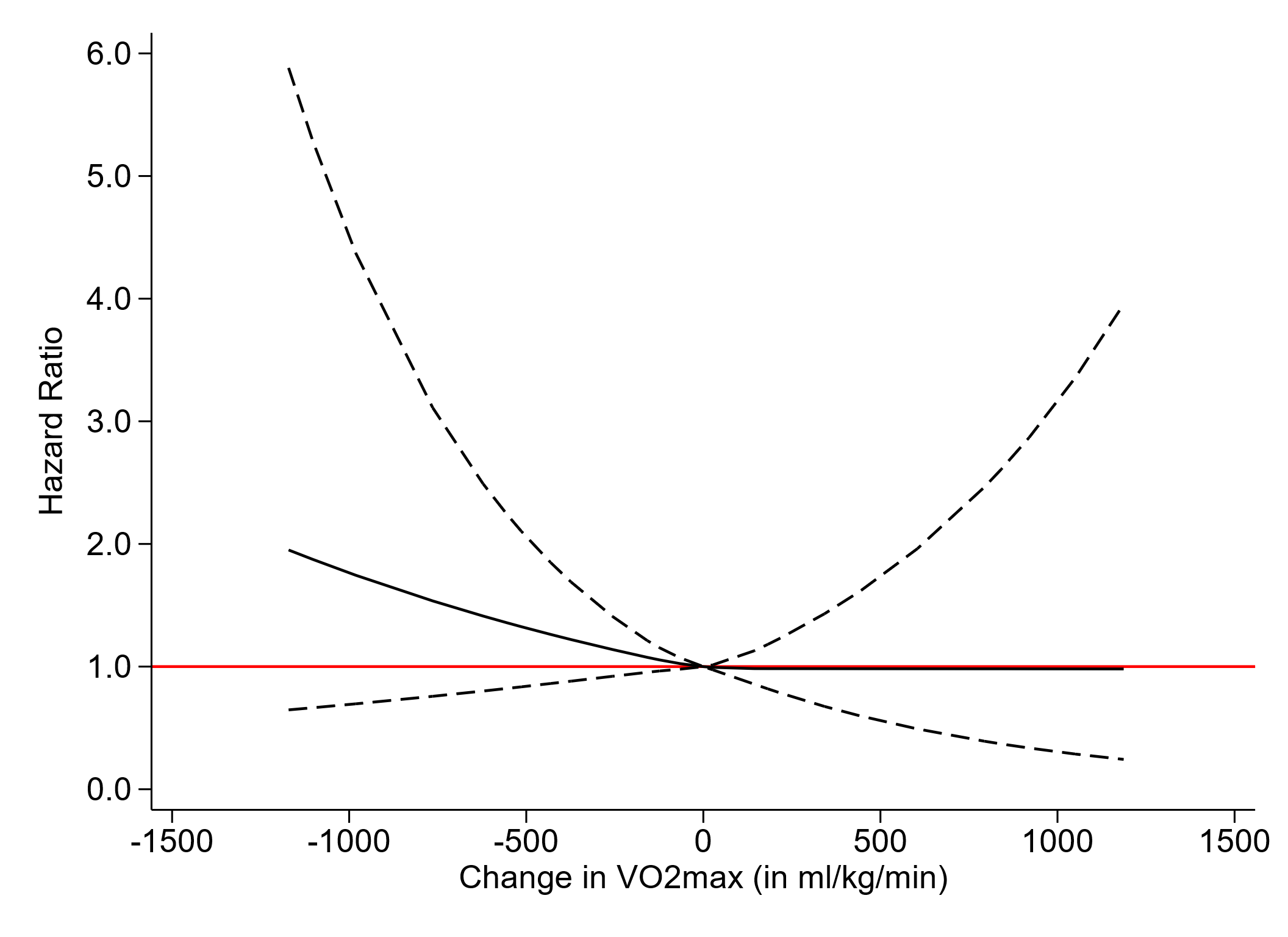


The solid black line represents the regression line. Dashed lines on either side of the solid black line show the 95% confidence interval (CI). The red line is for easy reference, and a 95%CI below or above the line is regarded as a meaningful association.

Adjusted for race, baseline age, smoking, body mass index, Townsend deprivation index, baseline systolic blood pressure, baseline diastolic pressure, use of statin, hypertension, diabetes, coronary heart disease, chronic obstructive pulmonary disease, stroke, atrial fibrillation, heart failure, myocardial infarction.

**Supplementary Figure 8c Association between changes in VO_2_max and the RDKF incidence identified using eGFR_CysC_ in females**


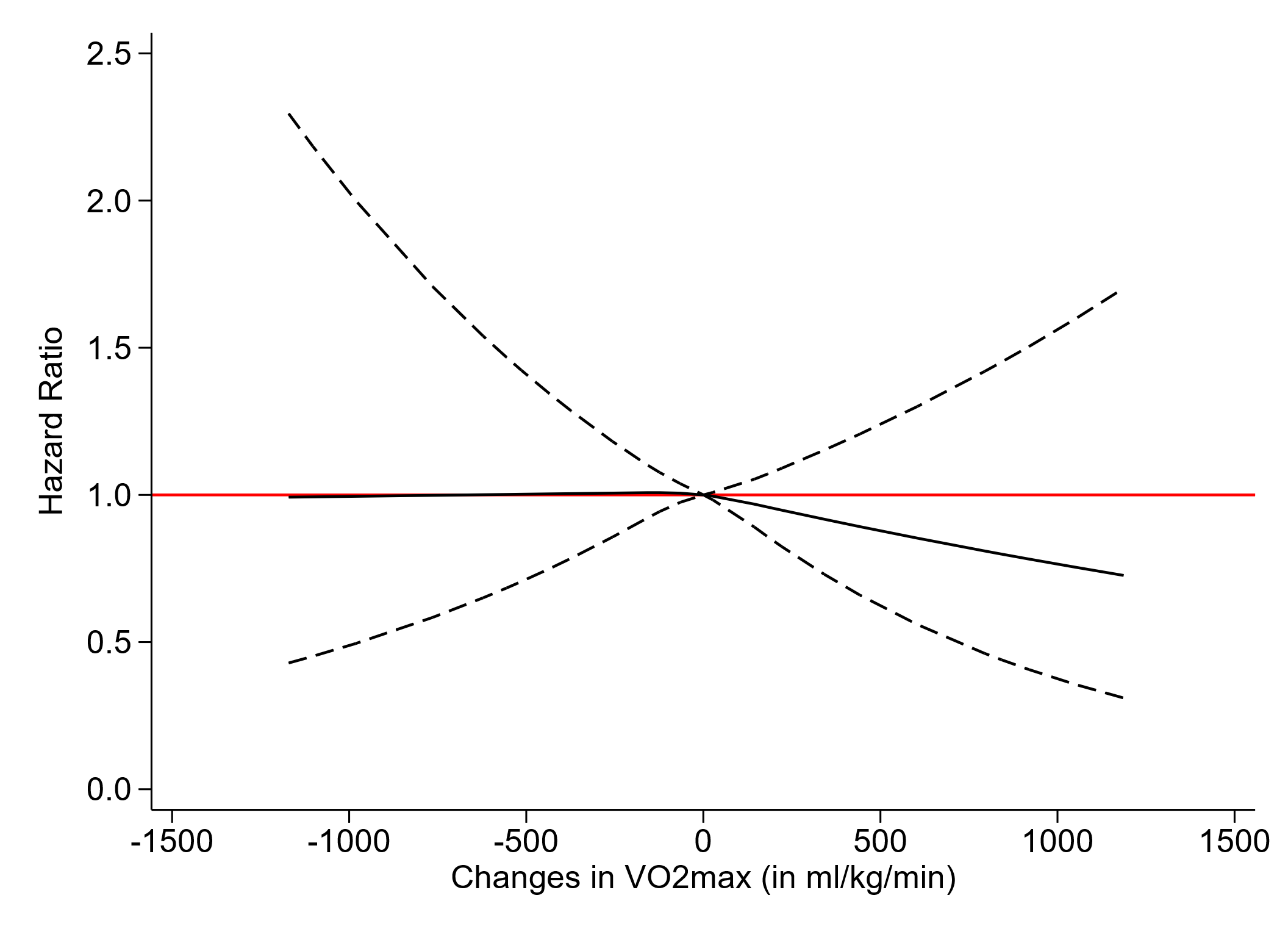


The solid black line represents the regression line. Dashed lines on either side of the solid black line show the 95% confidence interval (CI). The red line is for easy reference, and a 95%CI below or above the line is regarded as a meaningful association.

Adjusted for race, baseline age, smoking, body mass index, Townsend deprivation index, baseline systolic blood pressure, baseline diastolic pressure, use of statin, hypertension, diabetes, coronary heart disease, chronic obstructive pulmonary disease, stroke, atrial fibrillation, heart failure, myocardial infarction.

**Supplementary Figure 9a Association between changes in VO_2_max and the annual change of eGFR_SCr_**


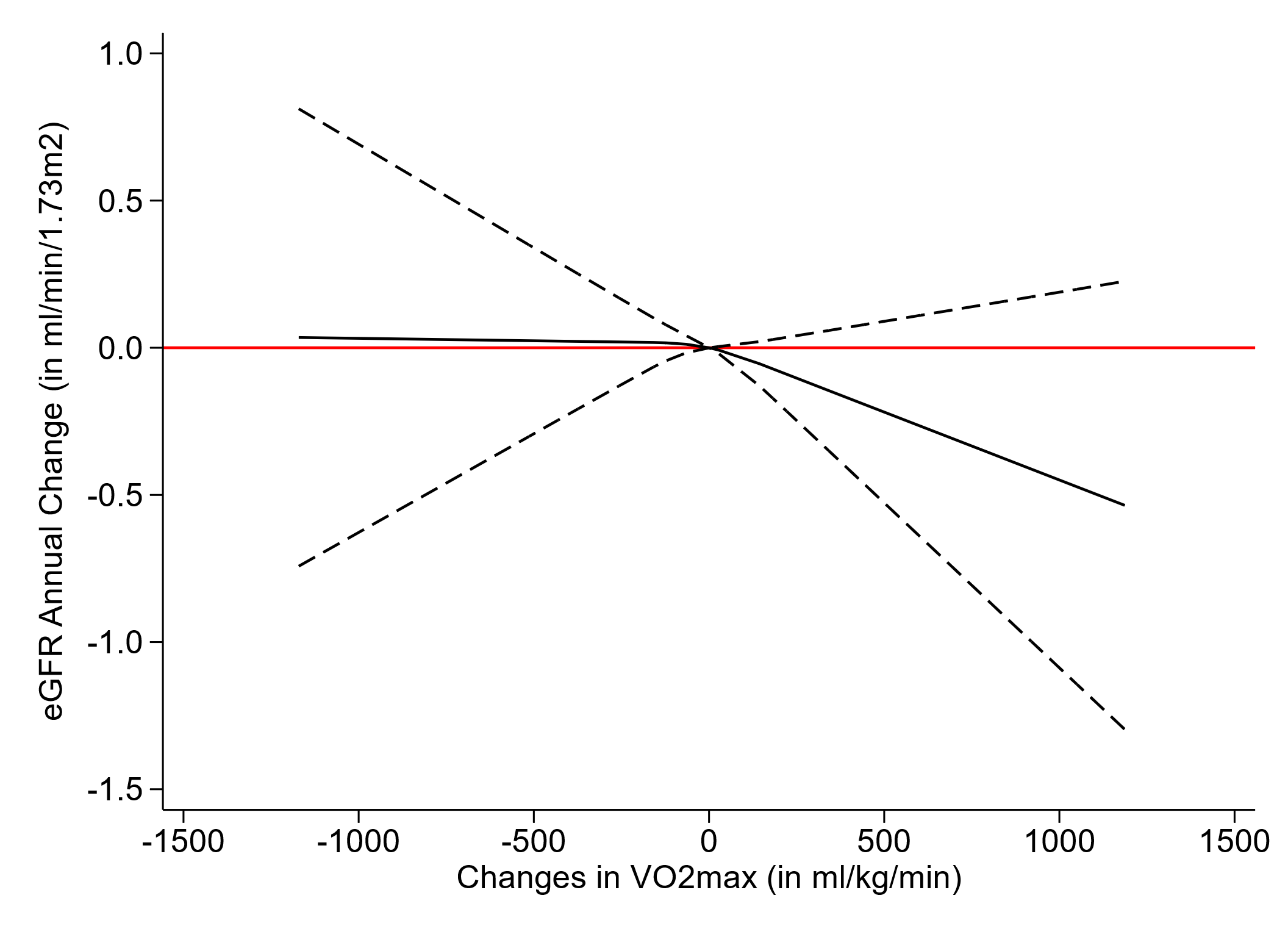


The solid black line represents the regression line. Dashed lines on either side of the solid black line show the 95% confidence interval (CI). The red line is for easy reference, and a 95%CI below or above the line is regarded as a meaningful association.

Adjusted for sex, race, baseline age, smoking, body mass index, Townsend deprivation index, baseline systolic blood pressure, baseline diastolic pressure, use of statin, hypertension, diabetes, coronary heart disease, chronic obstructive pulmonary disease, stroke, atrial fibrillation, heart failure, myocardial infarction.

**Supplementary Figure 9b Association between changes in VO_2_max and the annual change of eGFR_SCr_ in males**


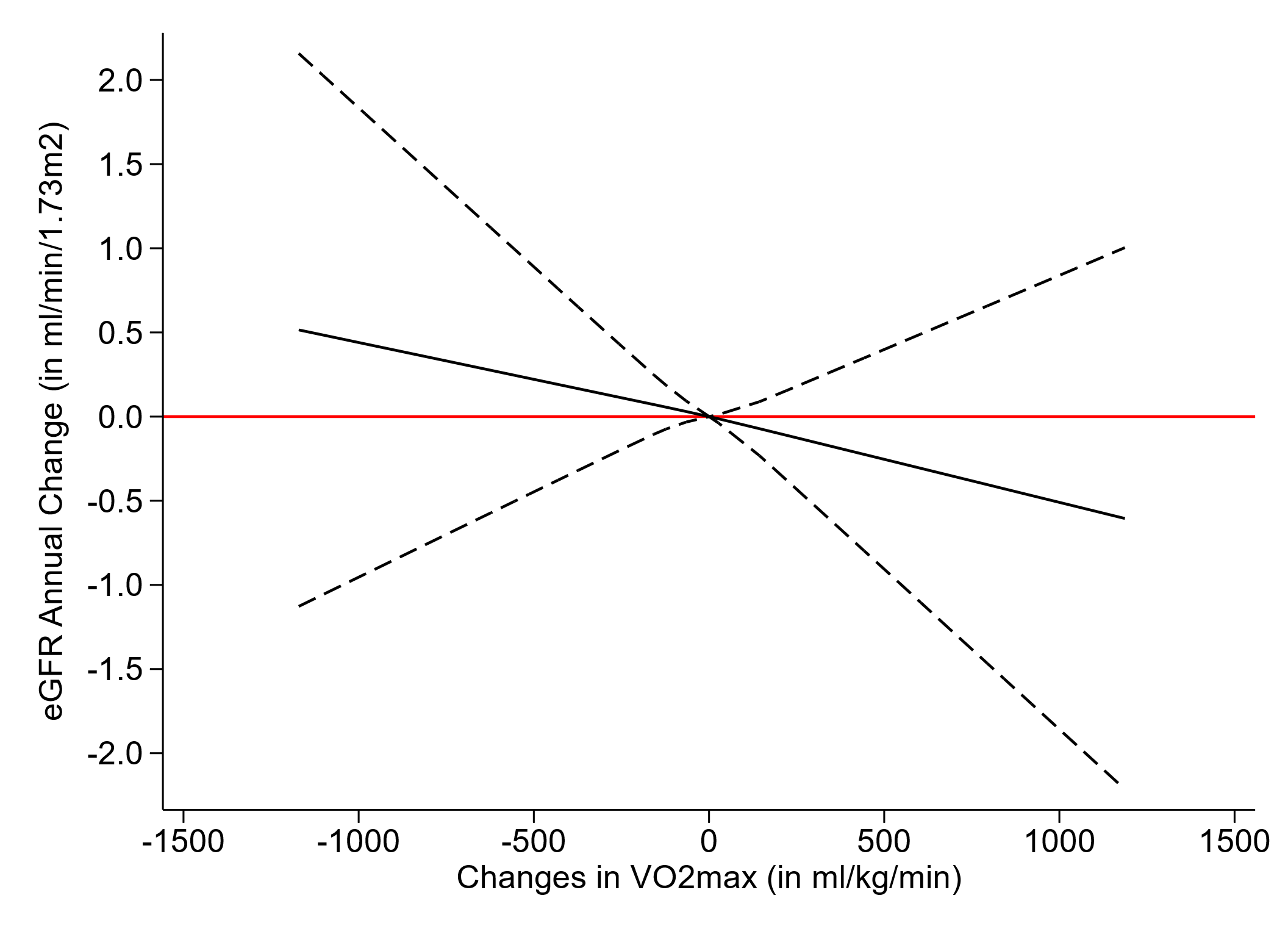


The solid black line represents the regression line. Dashed lines on either side of the solid black line show the 95% confidence interval (CI). The red line is for easy reference, and a 95%CI below or above the line is regarded as a meaningful association.

Adjusted for race, baseline age, smoking, body mass index, Townsend deprivation index, baseline systolic blood pressure, baseline diastolic pressure, use of statin, hypertension, diabetes, coronary heart disease, chronic obstructive pulmonary disease, stroke, atrial fibrillation, heart failure, myocardial infarction.

**Supplementary Figure 9c Association between changes in VO_2_max and the annual change of eGFR_SCr_ in females**


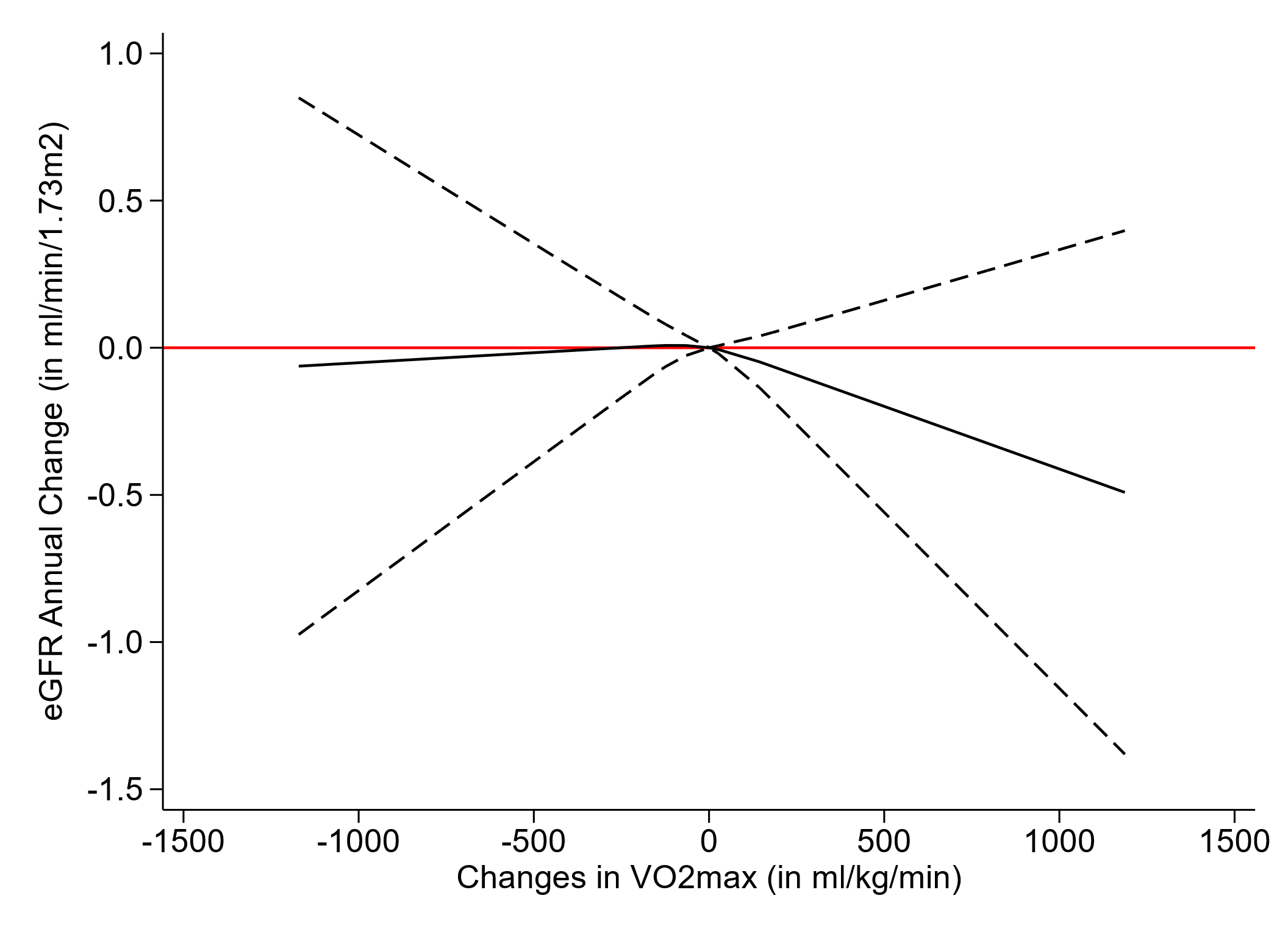


The solid black line represents the regression line. Dashed lines on either side of the solid black line show the 95% confidence interval (CI). The red line is for easy reference, and a 95%CI below or above the line is regarded as a meaningful association.

Adjusted for race, baseline age, smoking, body mass index, Townsend deprivation index, baseline systolic blood pressure, baseline diastolic pressure, use of statin, hypertension, diabetes, coronary heart disease, chronic obstructive pulmonary disease, stroke, atrial fibrillation, heart failure, myocardial infarction.

**Supplementary Figure 10a Association between changes in VO_2_max and the annual change of eGFR_CysC_**


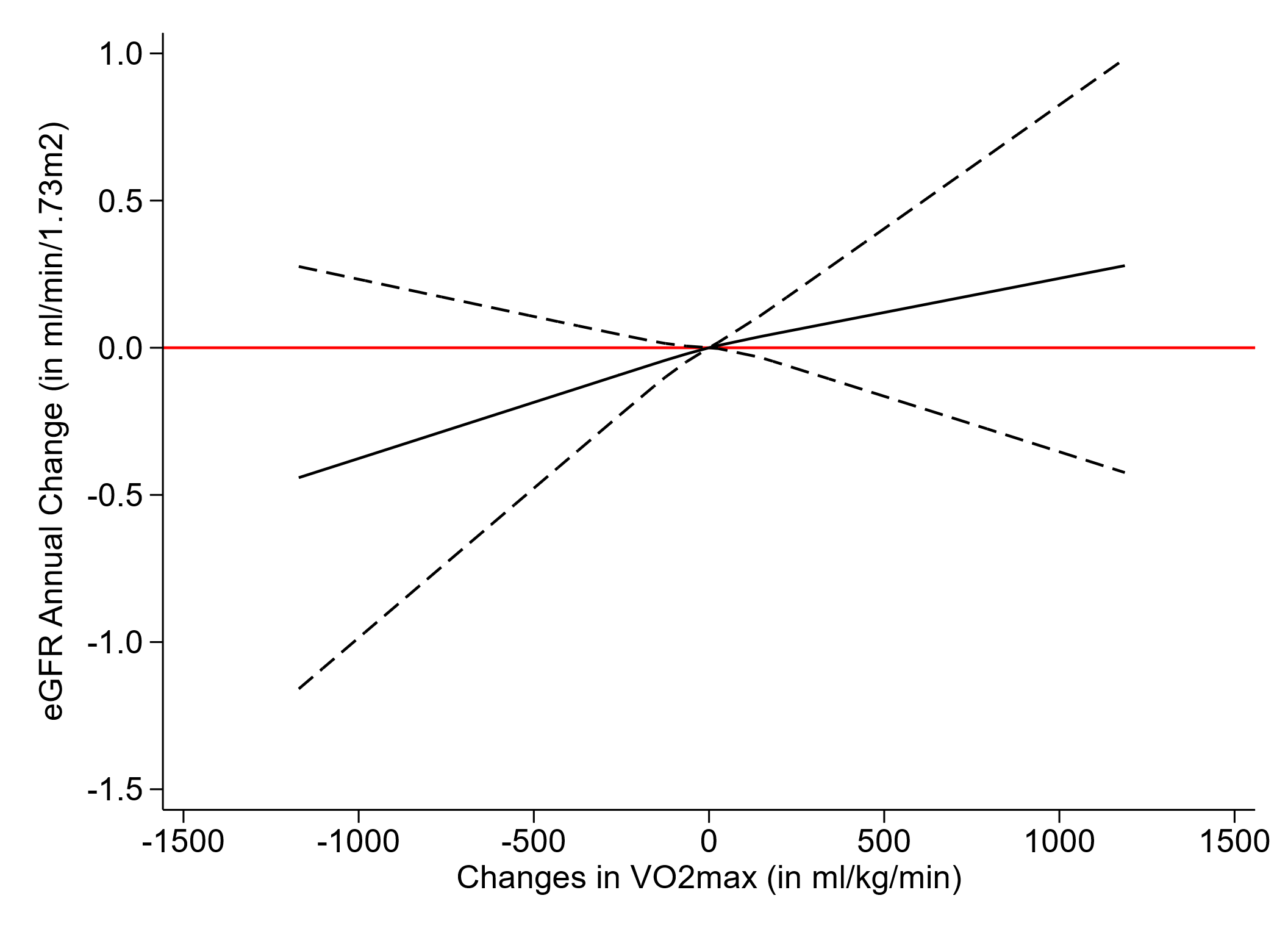


The solid black line represents the regression line. Dashed lines on either side of the solid black line show the 95% confidence interval (CI). The red line is for easy reference, and a 95%CI below or above the line is regarded as a meaningful association.

Adjusted for sex, race, baseline age, smoking, body mass index, Townsend deprivation index, baseline systolic blood pressure, baseline diastolic pressure, use of statin, hypertension, diabetes, coronary heart disease, chronic obstructive pulmonary disease, stroke, atrial fibrillation, heart failure, myocardial infarction.

**Supplementary Figure 10b Association between changes in VO_2_max and the annual change of eGFR_CysC_ in males**


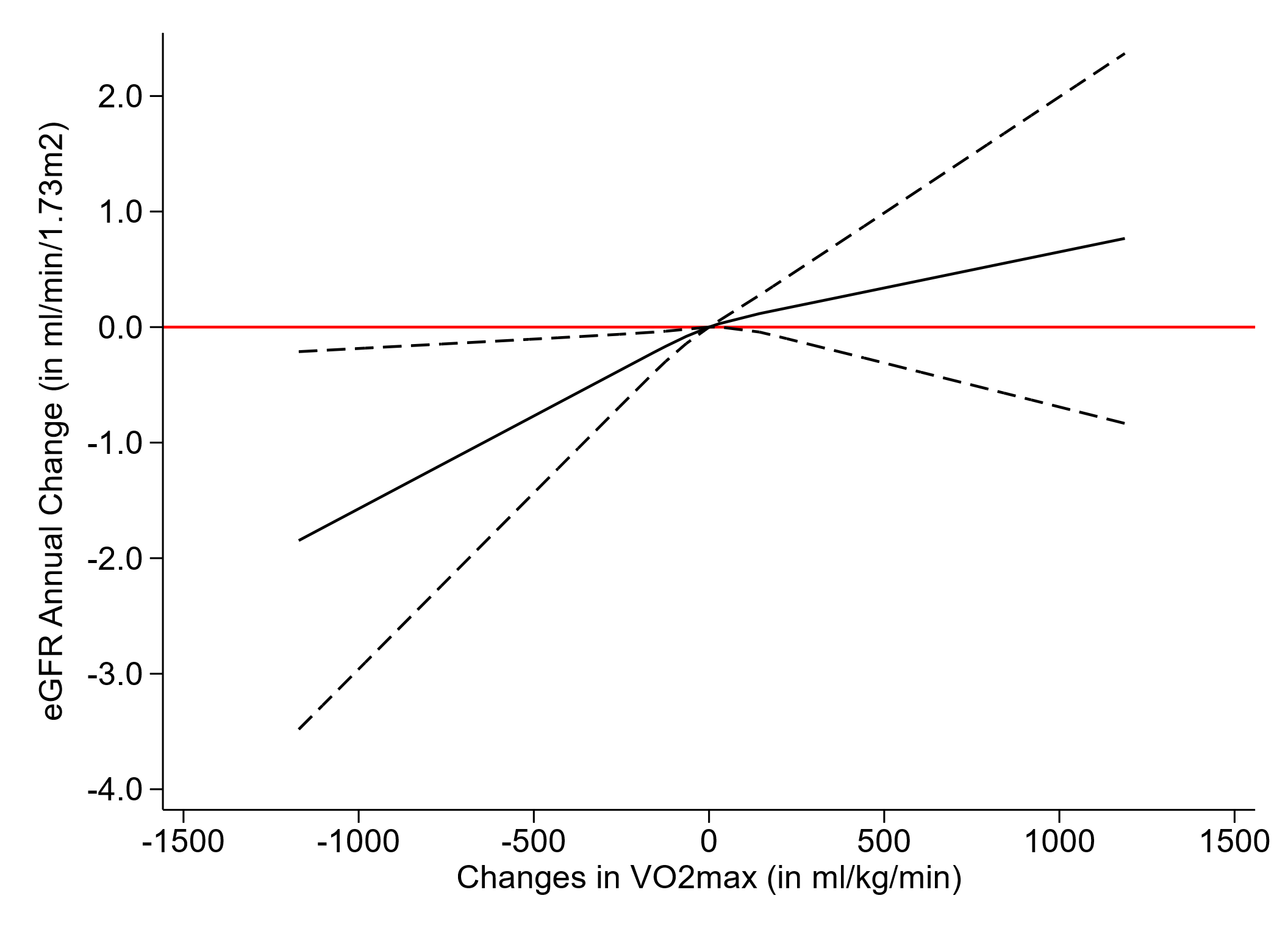


The solid black line represents the regression line. Dashed lines on either side of the solid black line show the 95% confidence interval (CI). The red line is for easy reference, and a 95%CI below or above the line is regarded as a meaningful association.

Adjusted for race, baseline age, smoking, body mass index, Townsend deprivation index, baseline systolic blood pressure, baseline diastolic pressure, use of statin, hypertension, diabetes, coronary heart disease, chronic obstructive pulmonary disease, stroke, atrial fibrillation, heart failure, myocardial infarction.

**Supplementary Figure 10c Association between changes in VO_2_max and the annual change of eGFR_CysC_ in females**


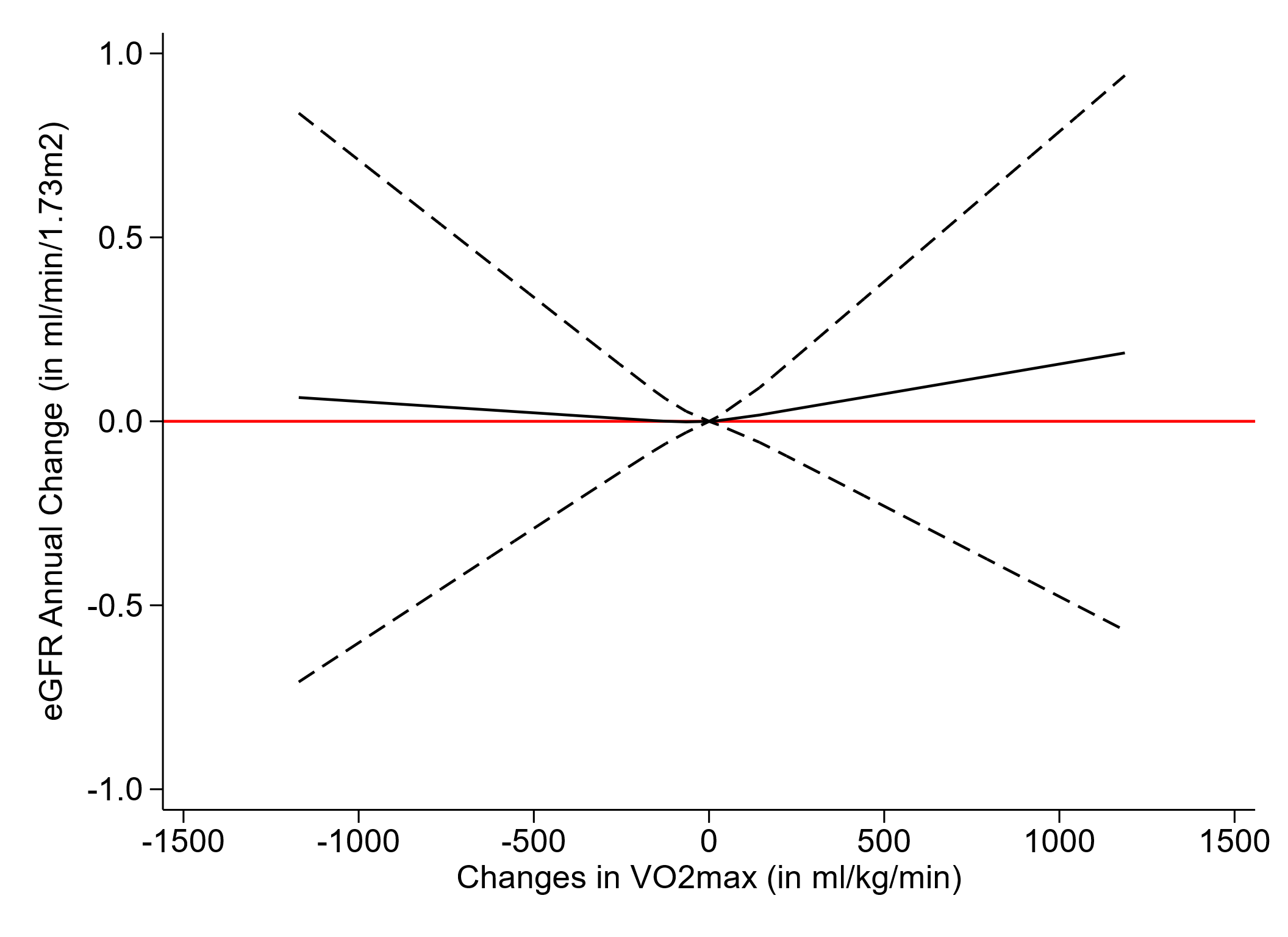


The solid black line represents the regression line. Dashed lines on either side of the solid black line show the 95% confidence interval (CI). The red line is for easy reference, and a 95%CI below or above the line is regarded as a meaningful association.

Adjusted for race, baseline age, smoking, body mass index, Townsend deprivation index, baseline systolic blood pressure, baseline diastolic pressure, use of statin, hypertension, diabetes, coronary heart disease, chronic obstructive pulmonary disease, stroke, atrial fibrillation, heart failure, myocardial infarction.
